# Fluorescent Tracers for *In Vivo* Imaging of Lymphatic Targets

**DOI:** 10.3389/fphar.2022.952581

**Published:** 2022-07-22

**Authors:** P. S. Russell, R. Velivolu, V. E. Maldonado Zimbrón, J. Hong, I. Kavianinia, A. J. R. Hickey, J. A. Windsor, A. R. J. Phillips

**Affiliations:** ^1^ Applied Surgery and Metabolism Laboratory, School of Biological Sciences, Faculty of Science, University of Auckland, Auckland, New Zealand; ^2^ Surgical and Translational Research Centre, Department of Surgery, Faculty of Medical and Health Sciences, University of Auckland, Auckland, New Zealand; ^3^ Maurice Wilkins Centre for Molecular Biodiscovery, School of Biological Sciences, Faculty of Science, The University of Auckland, Auckland, New Zealand; ^4^ School of Chemical Sciences, Faculty of Science, The University of Auckland, Auckland, New Zealand

**Keywords:** lymphatic, fluorescent imaging, *in vivo*, lymph node, fluorophore

## Abstract

The lymphatic system continues to gain importance in a range of conditions, and therefore, imaging of lymphatic vessels is becoming more widespread for research, diagnosis, and treatment. Fluorescent lymphatic imaging offers advantages over other methods in that it is affordable, has higher resolution, and does not require radiation exposure. However, because the lymphatic system is a one-way drainage system, the successful delivery of fluorescent tracers to lymphatic vessels represents a unique challenge. Each fluorescent tracer used for lymphatic imaging has distinct characteristics, including size, shape, charge, weight, conjugates, excitation/emission wavelength, stability, and quantum yield. These characteristics in combination with the properties of the target tissue affect the uptake of the dye into lymphatic vessels and the fluorescence quality. Here, we review the characteristics of visible wavelength and near-infrared fluorescent tracers used for *in vivo* lymphatic imaging and describe the various techniques used to specifically target them to lymphatic vessels for high-quality lymphatic imaging in both clinical and pre-clinical applications. We also discuss potential areas of future research to improve the lymphatic fluorescent tracer design.

## 1 Introduction

The lymphatic system functions as a one-way drainage system, returning interstitial fluid, macromolecules, and cells back into the systemic circulation, thereby fulfilling vital roles in fluid homeostasis, immune regulation, and dietary lipid absorption. Lymph fluid is initially formed in blind-ended lymphatic capillaries that lie within the interstitium throughout most of the body (exceptions include brain parenchyma, epidermis, bone marrow, and retina). These capillaries develop into larger collecting lymphatics that actively pump lymph towards lymph nodes (LNs), eventually entering the systemic circulation through the thoracic duct or right lymphatic duct. The most widely recognized consequence of lymphatic dysfunction is primary and secondary lymphedema (Rockson et al., 2019; Brouillard et al., 2021), but the lymphatic system has also been found to play a role in many other diseases, including cancer (Farnsworth et al., 2018), rheumatoid arthritis (Bouta et al., 2018), congestive heart failure (Fudim et al., 2021), peripheral vascular disease (Rasmussen et al., 2021), and inflammatory bowel disease (von der Weid et al., 2011). It is because of the emerging importance of lymphatic vessels that much attention has been devoted to methods of visualizing the lymphatic system *in vivo* and measuring its function.

Lymph is transparent, except within intestinal lymphatics that appear white after fat absorption, and so lymphatic vessels are not easily visible, even when viewed directly after surgical exposure (Munn and Padera, 2014). Therefore, visualizing lymphatic vessels *in vivo* usually requires a contrast agent. Because the lymphatic system is a one-way transport system in health, this also means that only the lymphatic vessels draining the injection site will be enhanced and not the entire lymphatic circulation (Munn and Padera, 2014). Many different lymphatic imaging techniques utilize contrast agents. Excluding fluorescent imaging, these include: 1) x-ray lymphangiography, involving direct cannulation of a peripheral lymphatic and infusion of a radiopaque contrast agent; 2) lymphoscintigraphy, involving an interstitial injection of radioactive tracer such as technetium-99m (^99m^Tc); 3) single-photon emission computed tomography (SPECT)/CT, combining lymphoscintigraphy with x-ray tomography to give more precise 3D localization of the radioactive signal; 4) magnetic resonance lymphography (MRL), where a commonly used tracer is ultrasmall super-paramagnetic iron oxide (USPIO); 5) direct visualization after vital blue dye injection; 6) contrast-enhanced ultrasound (CEUS), where gas-filled microbubbles act as contrast agents; 7) photoacoustic lymphangiography (PAL; also known as optoacoustic lymphangiography), which often uses indocyanine green (ICG) or methylene blue as a tracer and measures the reflected ultrasound waves (non-radiative decay, as opposed to fluorescence) dictated by the photoacoustic effect (Munn and Padera, 2014; Polomska and Proulx, 2021). All of these techniques have been used for a variety of indications, but perhaps the most common is sentinel lymph node (SLN; the first draining LN for a given anatomical site) mapping. Traditional SLN mapping and biopsy involves a peri-tumor injection of a radiocolloid and blue dye. The general area of the SLN is detected by a gamma detection probe, followed by skin incision, and identification and removal of the blue-colored node (Hameed et al., 2019).

Unlike the aforementioned methods, fluorescent lymphography (FL) involves exciting fluorophores within lymphatics or LNs at a certain wavelength and measuring the emitted light (radiative decay) as the fluorophore relaxes to a lower energy state. This may be either non-invasive (skin-intact) or invasive (surgically exposed) (Table 1). Advantages of fluorescent imaging over other methods include affordability, higher resolution, higher tissue penetration (compared to blue dyes), “invisibility” within the surgical field (for NIR dyes compared to blue dyes), improved signal-to-background ratio (SBR), technical ease, rapidity, real-time imaging, and absence of radiation exposure (Hameed et al., 2019; Polomska and Proulx, 2021). Because of these advantages, non-invasive FL, usually with ICG, has largely replaced lymphoscintigraphy in the investigation of lymphedema and other conditions (Unno et al., 2007) and is gaining widespread popularity in SLN detection (Hameed et al., 2019).

**TABLE 1:** Lymphatic fluorescent imaging can be broadly divided into non-invasive (skin intact) and invasive (surgically exposed) imaging. Non-invasive imaging normally requires a NIR tracer, unless only superficial skin lymphatics are viewed through a microscope (FML), or the tracer is particularly bright (Qdots). Surgically exposed lymphography can either use a NIR or visible wavelength fluorescent tracer.

Category	Description	Main tracers	Main uses
1) Non-invasive lymphography			
Near-infrared			
NIR fluorescent lymphography Unno et al. (2007); Sevick-Muraca et al. (2008); Proulx et al. (2010); Hutteman et al. (2011); Karlsen et al. (2012); Karaman et al. (2015); Yamamoto et al. (2016); Suami. (2017); Kajita et al. (2020b)	Intradermal/subcutaneous/peri-tumoral/etc. injection of NIR-I/II tracer, visualization of lymphatic vessels/nodes in NIR with a CCD camera or similar. NIR-II detection requires an InGaAs camera or similar	• ICG ± conjugate or delivery particle	1. Anatomy and function of lymphatics within available depth penetration of the tracer/imaging system
		• Alexa 680-albumin	2. SLN mapping
		• P20D800	3. Lymphatic flow in transplants (e.g., free flap, LN transfer)
		• Various nanoparticles	4. Identification of lymphatic vessels prior to LVA surgery
			5. Tissue tracer clearance or the rate of transport to LN
Visible wavelength			
Fluorescence microlymphography (FML) Leu et al. (1994); Veikkola et al. (2001); Padera et al. (2002a); Petrova et al. (2004); Bollinger and Amann-Vesti (2007); Keo et al. (2015)	Intradermal injection of the tracer. Superficial dermal lymphatics visualized with a microscope (e.g., multiphoton microscopy)	• FITC/TRITC-dextran	1. Diagnosis of limb lymphedema (*via* spread of the tracer)
			2. Animal anatomical studies
			3. Animal disease/genetically modified models
Quantum dot fluorescent lymphography (Kosaka et al. (2009))	Qdots can be bright enough to see through the skin at visible wavelengths	• Qdots	1. SLN mapping
2) Invasive lymphography			
Near-infrared or visible wavelength			
Surgically exposed fluorescent lymphography Isaka et al. (2004); Kassis et al. (2012); Moriondo et al. (2013); Liao et al. (2014); Johnson et al. (2020)	Surgical exposure of tissue of interest (no skin barrier), followed/preceded by the delivery of the tracer and image capture	• ICG	1. SLN mapping and LN transfer
		• FITC-dextran	2. Animal models (e.g., to study diaphragm, popliteal, mesentery, flank, and skin)
		• BODIPY	

CCD, charge-coupled device; FITC, fluorescein isothiocyanate; ICG, indocyanine green; InGaAs, indium gallium arsenide; LN, lymph node; LP-ICG, liposomal formulation of ICG; LVA, lymphovenous anastomosis; NIR, near-infrared; P20D800, 20 kDa PEG–IRDye800 conjugate; SLN, sentinel lymph node; TRITC, tetramethylrhodamine isothiocyanate.

FL also has limitations. Injection of a contrast agent can create a minor amount of tissue damage and alter interstitial pressure, having downstream effects on lymphatic pumping characteristics (Scallan et al., 2016). However, this is only problematic when precisely measuring lymphatic contractile parameters and is not usually an issue in a clinical setting when only very small doses of tracer are needed (Sevick-Muraca et al., 2008). For studies that require precise vessel diameter measurements, optical coherence tomography is a promising technique for visualizing lymphatics in animal models without using a contrast agent (Blatter et al., 2018), but it is limited to very superficial lymphatics (<2 mm below the tissue surface) (Polomska and Proulx, 2021). Another emerging lymphatic imaging technique that does not require a contrast agent is laser speckle imaging (Kalchenko et al., 2012), but this is limited by penetration depth, surface movement, and camera and post-processing requirements.

**FIGURE 1:**
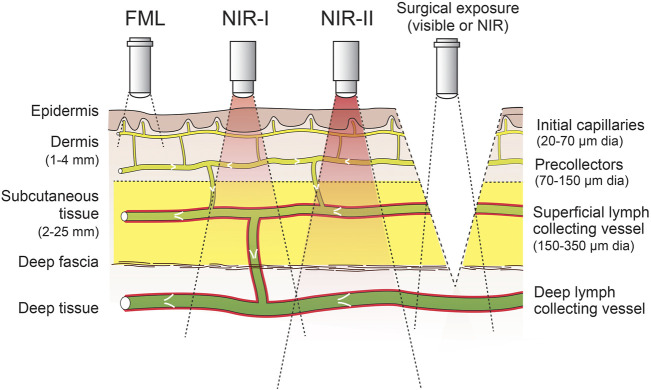
Use of fluorophores that emit at different wavelengths allows visualization of lymphatics at different depths for skin-intact imaging. Alternatively, surgical exposure can be used to visualize deeper lymphatic vessels. Intraluminal valves and smooth muscle cells are shown in white and red, respectively. Measurements on the left indicate approximate depths in human skin tissue, and measurements on the right indicate approximate diameters of lymphatic vessels. Fluorescence microlymphography (FML) uses fluorophores emitting at a visible wavelength (∼400–700 nm). The tissue depth at which NIR imaging devices can detect NIR fluorophores (NIR-I wavelength ∼700–1,000 nm; NIR-II wavelength ∼1,000–1,700 nm) is dependent upon their brightness and the sensitivity of the device, but it can be as much as 3–4 cm (Zhu and Sevick-Muraca, 2014).

The spatial resolution of FL, although superior to lymphoscintigraphy, can be seen as a further limitation in many instances. The limitation of spatial resolution can be improved by the use of PAL (Suzuki et al., 2020); however, this non-fluorescent technique is still under development and has important limitations itself, including poor depth penetration, interference from hair roots, and limited field of view (Kajita et al., 2020a). The limit in spatial resolution of FL, which relies on the detection of light radiation, is largely due to light-tissue interactions, which increase in complexity and significance at greater tissue depth. These interactions include interface reflection, in-tissue light scattering, in-tissue absorption, and tissue autofluorescence (Hong et al., 2017). The latter three factors can be decreased by the use of agents that fluoresce at longer wavelengths, which allows imaging at greater depths (Figure 1) (Hong et al., 2017). When wavelength boundaries are inconsistent in the literature, for the purpose of this review, we defined the visible wavelength range as 400–700 nm, near-infrared (NIR)-I as 700–1,000 nm, NIR-II as 1,000–1,700 nm, and short-wavelength infrared (SWIR) as 1,700–3,000 nm based on Hong et al. (2017), Liu et al. (2021). While tissue autofluorescence increases as excitation wavelengths shorten (Zhu and Sevick-Muraca, 2014), water strongly absorbs light at wavelengths above 900 nm, and therefore an optical window for *in vivo* imaging is present between 650 and 900 nm (Miwa, 2016) with further optical windows in the NIR-II range, which are discussed later.

Despite the limitations of light-tissue interactions, both visible and NIR lymphatic fluorophores are widely used for a range of pre-clinical and clinical indications, as outlined in Table 1. The goal of lymphatic fluorescent imaging is to optimize the brightness [absorption coefficient (*ε*) × quantum yield (*ϕ*_fl_)] of the fluorescent signal from the lymphatic vessel compared to the background while retaining as much spatial resolution as possible. All lymphatic fluorescent tracers have certain properties that make them suitable for lymphatic uptake, and many studies have aimed to further optimize those tracers to better achieve this goal. The preferred fluorescent tracer depends on the indication for use and the tissue being imaged. Because of the importance of FL in both clinical and research settings, and because of the wide array of available fluorophores, the aim of this review is to describe the characteristics of fluorescent tracers that allow for optimum lymphatic imaging. With this aim in mind, an overview of interstitial and lymphatic anatomy is given, followed by a discussion of tracer administration routes for lymphatic uptake. Lymphatic-targeted fluorescent tracers are then discussed from visible to NIR-II wavelengths. Although the emphasis is on tracer characteristics, successful FL depends crucially on the imaging device used, and so this will also be briefly described with the relevant fluorescent tracers. The review is concluded with possible future research directions. As the focus of this review is *in vivo* lymphatic imaging of fluorescent dyes, histological fluorescent staining of lymphatics, green fluorescent protein (GFP)-expressed lymphatic vessels or cells, and non-fluorescent dyes are outside its scope. There is also a wide variety of cancer cell-targeted fluorescent tracers that can be used to visualize metastatic LNs. While not specifically lymphatic targeted and therefore not comprehensively reviewed, we mention specific examples to highlight the range of methods available to visualize LNs.

### 1.1 Anatomy of the Interstitium and Lymphatics

The interstitium includes all connective and supporting tissues located outside blood and lymphatic vessels and parenchymal cells (Wiig and Swartz, 2012). It consists of the interstitial fluid and the extracellular matrix (Wiig and Swartz, 2012). Interstitial fluid is taken up into initial lymphatics to form lymph, and therefore lymphatic-targeted fluorescent tracers, unless infused directly into lymphatic vessels or nodes, must move from the interstitium into initial lymphatics. The movement and subsequent lymphatic uptake of a tracer molecule partly depend on a molecule’s ability to move through the interstitium. *Hydraulic conductivity*, defined as the speed of fluid movement through the interstitium for a given pressure gradient, is the primary interstitial property that determines this (Wiig and Swartz, 2012). This property differs across tissue types (see Swartz and Fleury (2007) for details). For example, it is relatively high in mouse tail skin (70–150 cm^2^/mmHg s) (Swartz et al., 1999) but lower in rat dermis (5.33 cm^2^/mmHg s) (Bert and Reed, 1995). It is also dependent on tissue hydration, with much higher hydraulic conductivity seen in edematous tissue (Wiig and Swartz, 2012). Other interstitial properties that affect tracer movement include charge, composition, and compliance (Wiig and Swartz, 2012).

The revised Starling principle greatly contributed to the understanding of fluid movement within the interstitium, and between the interstitium, capillaries, and lymphatics (Levick and Michel, 2010). This principle (based on experimental and theoretical evidence) dictates that capillary filtration occurs the whole way along the microvascular bed in most tissues under steady-state, as opposed to the traditional view that capillary absorption occurs at the venous end of microvascular beds. The reason for this is that interstitial colloid osmotic pressure (COP) (one of four Starling forces that dictate fluid movement) itself varies with varying rates of fluid flux from the capillary, so even if capillary hydrostatic pressure fell enough to favor absorption, any transient absorption will decrease the difference between capillary and interstitial COP until steady-state (slight filtration) is again reached. The implication of this for the lymphatic system is that lymphatics, rather than veins, are almost exclusively responsible for the recirculation of fluids and macromolecules from the interstitium (Mortimer and Levick, 2004). Notable exceptions are the bowel wall, kidney, and LNs, where the interstitial fluid is continuously replaced by epithelial secretion (bowel and kidney) or low-protein-concentration lymph fluid (LNs), keeping interstitial COP low and favoring absorption into blood capillaries. An injection of fluid (such as a fluorescent tracer) into the interstitium resembles these latter scenarios in that it can acutely raise interstitial hydrostatic pressure, decrease interstitial COP, and cause transient capillary absorption. For completion, it should be noted that theoretical capillary filtration was initially calculated to be much higher than the measured lymph flow from most tissues. This discrepancy was addressed by the realization that the endothelial glycocalyx was the important semi-permeable membrane between the capillary and interstitium, and the sub glycocalyx protein concentration (within the endothelial intercellular clefts) was lower than the bulk interstitial protein concentration, therefore resulting in much less capillary filtration than previously calculated (Levick and Michel, 2010).

The normal fluid movement from the blood capillary to the initial lymphatic capillary creates a convective flow within the interstitium towards the initial lymphatics (Trevaskis et al., 2015). The major mechanism of uptake into the initial lymphatic capillary is thought to be *intermittent hydraulic pressure gradients*, where pressure within the initial lymphatic temporarily drops below interstitial pressure to allow passive movement into the lymphatic (Wiig and Swartz, 2012). The effectiveness of intermittent hydraulic pressure gradients relies on the presence of primary valves, formed by the overlapping endothelial cells, that prevent fluid escape from the initial lymphatic when the pressure gradient is reversed (Schmid-Schönbein, 2003). It also relies on anchoring filaments, composed primarily of fibrillin, which anchor the endothelial cells to the surrounding extracellular matrix (Gerli et al., 2000) and are thought to help maintain vessel patency against an adverse pressure gradient and open primary valves to allow inward flow (Wiig and Swartz, 2012). *Active transport* across endothelial cells is also likely to play a role (Trevaskis et al., 2015), even including uptake of water *via* aquaporin-2 on the surface of lymphatic endothelial cells (Miteva et al., 2010). Active transport is therefore also likely to play a role in fluorescent tracer uptake.

Initial lymphatic vessels are in continuity with pre-collectors, characterized by the presence of intraluminal valves but without lymphatic muscle, and are themselves in continuity with collectors, characterized by intraluminal valves and a layer of lymphatic muscle (Breslin et al., 2018). The contractile unit of a collecting lymphatic vessel is the section between two intraluminal valves, called the lymphangion, which is surrounded by a layer of lymphatic muscle (Scallan et al., 2016). One or more afferent collecting vessels then enter a LN, which is designed for efficient interaction of antigen-presenting cells with lymphocytes (Willard-Mack, 2006). The composition of lymph is altered in the LN, largely due to the influx of lymphocytes through high endothelial venules (Willard-Mack, 2006). This lymph exits the LN *via* the efferent lymphatics (that also contain a layer of muscle and intraluminal valves), which coalesce into the main transport lymphatics of the body, such as the thoracic duct.

### 1.2 General Aspects of Lymphatic Targeting

**FIGURE 2:**
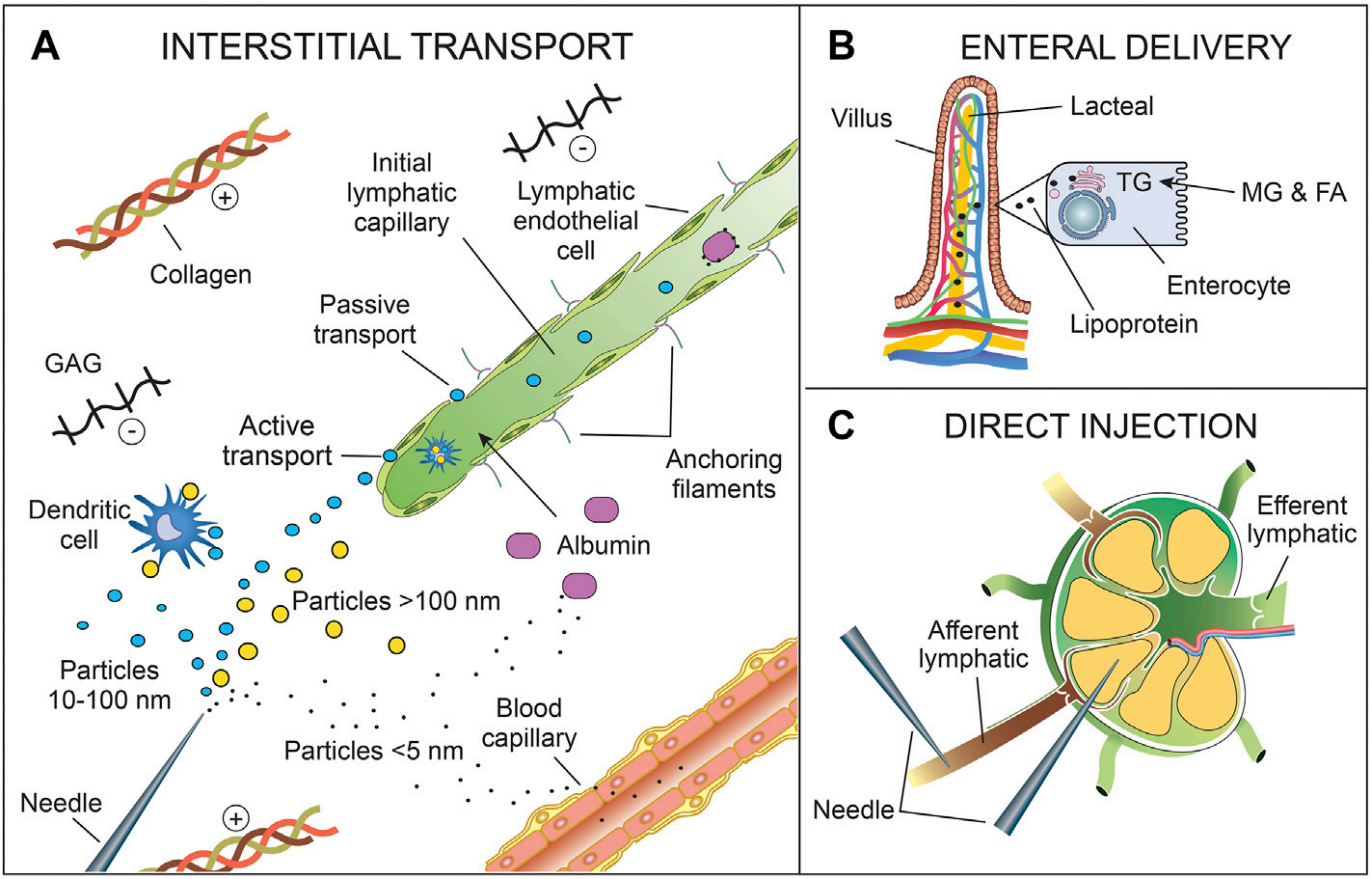
Delivery routes for lymphatic uptake of fluorescent tracers. **(A)** Interstitial transport includes intradermal, subcutaneous, submucosal, peritumoral, or intraparenchymal injection. Other delivery routes (enteral, endotracheal, intra-articular, intravascular) also include interstitial transport prior to lymphatic uptake. The tracers enter lymphatic vessels either singly or bound to interstitial cells (e.g., dendritic cells) or macromolecules (e.g., albumin) *via* passive transport between endothelial cells or active transport across endothelial cells. Particles >10 nm (or >20 kDa) (blue) are generally taken up by the lymphatics, as opposed to the blood capillaries. Particles >100 nm (or >30 kDa) (yellow) are still taken up by lymphatics but diffuse poorly through the interstitium. Particles <5 nm (or <20 kDa) (black dot) preferentially enter blood capillaries if they remain unbound. **(B)** Enteral delivery of fluorescent tracers to mesenteric lymphatics can be achieved by using a FA fluorescent tracer (e.g., BODIPY) or conjugating the tracer to a FA. Dietary triglycerides are broken down into FAs and 2-MG by pancreatic lipase prior to absorption. Similarly, lipophilic fluorescent tracers need to be small FAs or stable in the gastrointestinal tract to prevent enteric breakdown. Once absorbed, FAs (>10 carbon atoms in length), including fluorescent FAs, are reesterified into TGs and then incorporated into lipoproteins within the enterocytes. Lipoproteins, primarily chylomicrons, are preferentially taken up in the lacteal. **(C)** Fluorescent tracers can be injected directly into lymphatic vessels or nodes. Although not shown here, the tracer may enter the LN through intra-nodal high endothelial venules after intravenous injection. Also not shown is intraperitoneal delivery. Abbreviations: FA, fatty acid; GAGs, glycosaminoglycans; MG, 2-monoglyceride; TG, triglyceride.

The ability of a fluorescent tracer to move within the tissue and enter initial lymphatics is dependent on tracer characteristics, such as size, charge, and protein binding, which can be manipulated to enhance lymphatic uptake. Of these, *size* is perhaps the most important for lymphatic targeting because anything too small (typically <5 nm diameter, or <10 kDa) can rapidly diffuse throughout the interstitium and enter blood vessels (Figure 2), where preferential clearance of the tracer *via* blood occurs because blood flow is 100–500 times greater than lymph flow (Polomska and Proulx, 2021). Alternatively, anything too large (typically > 100 nm) diffuses poorly through the interstitium and remains mostly at the injection site (Trevaskis et al., 2015; Rohner and Thomas, 2017). It is generally agreed that 10–100 nm or 20–30 kDa is the optimal size for lymphatic uptake (Trevaskis et al., 2015). Increased size can also be important for preventing collecting lymphatic vessel leaks once inside the lymphatic vessel.

Increasing the effective size of the fluorophore, such as by conjugating to dextran, albumin, or PEG, is a common method of lymphatic targeting (Abdallah et al., 2020). Polyethylene glycol (PEG) is a hydrophilic polyether compound with the general structure H–(O–CH_2_–CH_2_)_n_–OH. Considered safe by the Food and Drug Administration (FDA), it has found many uses in medicine and other industries. As well as increasing the size of a molecule, PEGylation decreases interstitial interactions and immunogenicity by providing a steric barrier, reducing phagocytosis and therefore excessive long-term LN retention (Trevaskis et al., 2015). The hydrophilic nature of PEG also improves solubility. The degree of lymphatic uptake can vary with the size of the PEG molecule. Kaminskas et al. (2009) showed that increasing the PEG length from 200 to 2,000 Da greatly enhanced the lymphatic uptake of PEGylated poly-L-lysine dendrimers after subcutaneous injection, and Doan et al. showed venous and lymphatic uptake of 2- and 40-kDa PEG, respectively, conjugated to IRDye800CW after intra-articular injection into the rat knee joint (Doan et al., 2019). Proulx et al. also showed that a 20 kDa PEG chain improved lymphatic specificity, while still remaining small enough for easy diffusion through the skin (Proulx et al., 2013).

Another common method of increasing the effective fluorophore size is incorporation into a larger particle. Nanoparticles (constructs with an overall dimension below 100 nm) are commonly used for this and can be modified in highly tunable ways (Murthy, 2007). Other larger particles are liposomes (lipid bilayers) and polymer microspheres. Liposomes are also often commonly coated with PEG, which has been shown to further enhance lymphatic uptake (Lee et al., 2019). The size of the particle can also have a significant effect on the movement of the tracer within the lymphatic vasculature. For example, liposomes (and other molecules >70 kDa in size) are confined to LN sinuses due to their size (Gretz et al., 2000).

The *charge* of a tracer also affects its distribution and lymphatic uptake, but it is unclear whether the interstitium typically carries a net positive or negative charge, and this may vary between tissues over time. In the interstitium, glycosaminoglycans (GAGs) carry negative charges, however, collagen, which is far more abundant, carries a slightly positive charge (Wiig and Swartz, 2012). This may result in a slight net positive charge in the interstitium, at least in subcutaneous tissue (Gilányi and Kovách, 1991). Moreover, negatively charged GAGs can also attract positively charged tracers and influence tracer distributions (Park et al., 2016). The complexity of charges within the interstitium goes some way toward explaining the ambiguity in the literature in regard to the effect of tracer charge on the interstitial distribution of the tracer (Polomska and Proulx, 2021).

In general, lymphatic targeting can be specific or non-specific. The vast majority of FL relies on non-specific fluorophores (e.g., ICG) that are taken up into the lymphatic vasculature along with the other mobile components of the interstitium. Specific targeting of fluorophores relies on molecular interactions between the fluorophore and the cells of lymphatic vessels or nodes, or cells within the lymphatic vessels or nodes, such as cancer cells. Non-specific fluorophores cannot detect whether the SLN contains metastatic cells or not, unlike specific fluorophores, which is an area of increasing research.

### 1.3 Administration Route

Like interstitial and fluorescent tracer properties, lymphatic fluorescent imaging depends crucially on the administration route of the fluorescent tracer (Figure 2). Gaining access to lymphatic vessels can only occur either by direct infusion into a lymphatic vessel or node (Bachmann et al., 2019) or by indirect access through the interstitium, followed by uptake into initial lymphatics. The latter is the more common delivery route, especially in a clinical setting where direct cannulation of lymphatic vessels is time-consuming, damaging, and invasive. Indirect access through the interstitium includes conventional injection techniques (e.g., intradermal (Bollinger and Amann-Vesti, 2007), subcutaneous (Suzuki et al., 2020), submucosal (Parungo et al., 2005), intratumoral (Tian et al., 2020), intraparenchymal (Soltesz et al., 2005)), administration into a body cavity prior to interstitial uptake across an epithelial layer (e.g., enteral (Kassis et al., 2012), endotracheal (Outtz Reed et al., 2020), and intra-articular (Doan et al., 2019)), or even intravascular administration followed by extravasation into the interstitium (Kaminskas et al., 2009). Intravascular administration may also be used to deliver tracers to lymph nodes *via* transport across high endothelial venules within the node. Intraperitoneal administration is different as the peritoneal cavity is in direct continuity with lymphatic vessels *via* lymphatic stomata (1.59–1.82 µm in diameter in humans (Ji-Chang and Shou-Min, 1991)), which are most abundant in the muscular diaphragm, and therefore intraperitoneal fluorescent tracers do not need to cross an epithelial or mesothelial layer to gain access to lymphatics (Isaza-Restrepo et al., 2018; Lee et al., 2019). Pleural stomata also directly connect parietal pleural lymphatics with the pleural space (Solari et al., 2022). Intrathecal (intraventricular or intracisternal) delivery of fluorescent agents has also been used to study lymphatics draining the cerebrospinal fluid (Ma et al., 2017).

For imaging skin lymphatics, intradermal or Mantoux injections are usually preferred over subcutaneous injections because the high pressure generated from the dense collagen matrix of the dermis aids in driving the injected fluid into the initial lymphatics (Harvey et al., 2011). Furthermore, the density of initial lymphatics is greater in the dermis, which includes both a superficial and deep dermal layer (Figure 1) (Richter and Jacobsen, 2014). Intradermal injections are usually painful, but this can be avoided by the use of microneedles that allow painless delivery to a specific depth [reviewed in Polomska and Proulx (2021)]. There is also some evidence that lymphatic uptake can be minor (∼3%) after subcutaneous injection (Kagan et al., 2007).

Mesenteric lymphatics and the downstream cisterna chyli and thoracic duct can be visualized by enteral delivery of a fatty acid fluorescent tracer (e.g., BODIPY) or a fatty acid-conjugated tracer because fatty acids derived from dietary triglycerides are normally incorporated into chylomicrons in the bowel wall and taken up by lymphatics (Trevaskis et al., 2015). This is a useful administration route for a range of indications, including delivery of fluorescent contrast and therapeutic agents, because these lymphatics are not easily accessed otherwise, and if this route can be used for a certain indication, it obviates the need for a painful injection. To date, lymphatic-targeted enteral delivery of a fluorescent contrast agent remains a pre-clinical method.

The characteristics of various fluorophores that can be optimized for improved lymphatic uptake and imaging will now be discussed in order of increasing emission wavelength.

## 2 Visible Wavelength Fluorophores: Green

Due to the high absorption of visible light by water, blood, and melanin (Sharma et al., 2008), visible wavelength (400–700 nm) fluorescent dyes are generally only used when the lymphatic vessel lies superficially. In practice, this either involves superficial dermal lymphatics visualized with a microscope or surgically exposed tissue (e.g., axilla, groin, mesentery, popliteal region). The main advantages of using visible wavelength dyes are the increased resolution and ability to image without specialized NIR detectors. The primary methods of lymphatic targeting of visible wavelength fluorophores are conjugation to dextran, incorporation into polystyrene microspheres, enteral delivery of a fluorescent fatty acid, and fluorescently labeled antibodies or cells.

### 2.1 Conjugation to Dextran or Albumin

The most commonly used visible wavelength dye for visualizing lymphatics *in vivo* is fluorescein isothiocyanate (FITC) (ex/em 495/519 nm), a xanthene-derived fluorescent organic dye (fluorescein) linked to a reactive isothiocyanate group, which reacts with amine and sulfhydryl groups on proteins (Table 2). However, because of its small size (∼390 Da), it is conjugated to the anhydroglucose polymer dextran for lymphatic imaging. Dextran is available in a range of sizes, generally from a molecular weight of 3 kDa up to 2,000 kDa. As mentioned earlier, 20–30 kDa dextrans are optimum for transport to the lymphatics from the interstitium, but larger dextrans are often used to minimize lymphatic leak (Padera et al., 2002a; Bollinger and Amann-Vesti, 2007). Dextrans have been shown not to bind to plasma proteins (Rutili and Arfors, 1972) but FITC-dextran undergoes relatively rapid photobleaching (fluorescence half-life of 19 s under continuous illumination in anhydrous glycerol media) (Benchaib et al., 1996).

**TABLE 2:** Common fluorescent tracers used for lymphatic applications.

Fluorophore	Properties	Structure	Phase	Examples of conjugates/particles for lymphatic imaging
Fluorescein isothiocyanate (FITC)	MW = 390 Da λ_abs/em_ = 495/519 *ϕ* = 92% (PBS) *ε* = 73,000 M^−1^ cm^−1^	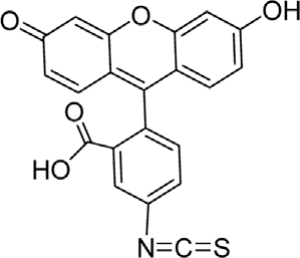	FDA-approved	- FITC-dextran (150–2,000 kDa) (intradermal delivery for FML)
				- FITC microspheres (intraperitoneal delivery for FL of diaphragmatic lymphatics)
BODIPY® FL C16	MW = 474 Da	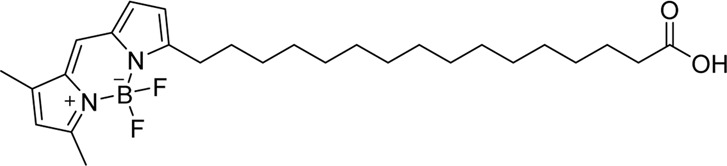	Pre-clinical	Fluorescent fatty acid used for visualizing the mesenteric lymphatic or thoracic duct after enteral delivery
	λ_abs/em_ = 490/520			
	*ϕ* = 100%			
	*ε* = 80,000 M^−1^ cm^−1^			
Cy5	MW = 1,051 Da λ_abs/em_ = 649/666 *ϕ* = 18% (PBS) *ε* = 250,000 M^−1^ cm^−1^	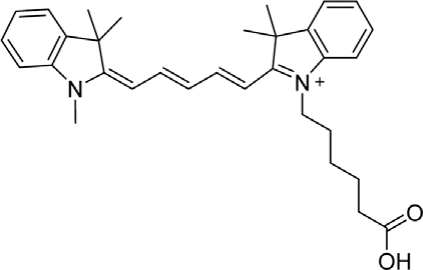	Pre-clinical	- Cy5-NHS-capric acid NEM (enteral delivery for pancreatic drug delivery)
				- Cy5-B_12_ (intradermal delivery for SLN mapping)
Cy5.5	MW = 716 Da λ_abs/em_ = 675/694 *ϕ* = 24% (PBS) *ε* = 250,000 M^−1^ cm^−1^	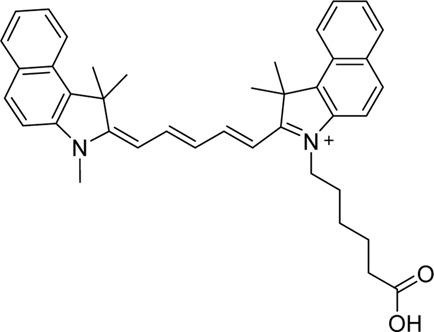	Pre-clinical (although cRGDY-PEG-Cy5.5-C dots in phase II study)	- Cy5.5-Rituximab (subcutaneous delivery for SLN mapping)
				- IgG-Cy5.5 (intradermal delivery for SLN mapping)
				- Cy5.5-PGC (subcutaneous/intravenous delivery for enzymatically activated SLN mapping)
				- cRGDY-PEG-Cy5.5-C dots (intradermal delivery for SLN mapping)
Indocyanine green (ICG)	MW = 775 Da λ_abs/em_ = 775/830 *ϕ* = 0.5% (water) *ε* = 262,100 M^−1^ cm^−1^	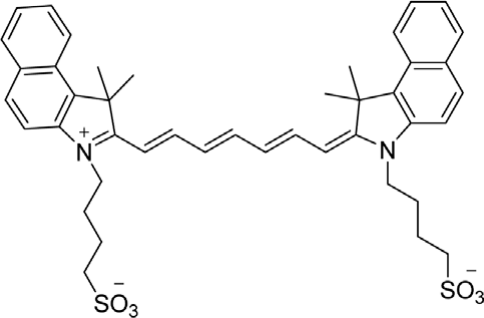	FDA approved	- ICG-albumin (intradermal delivery for FL/SLN mapping)
				- LP-ICG (intradermal delivery for FL/SLN mapping)
				- ICG-Kolliphor HS15 (intradermal delivery for FL/dermal lymphatic function imaging)
				- ICG-C11 (intradermal delivery for FL/SLN mapping)
Cy7	MW = 627 Da λ_abs/em_ = 753/775 *ϕ* = 28% (PBS) *ε* = 250,000 M^−1^ cm^−1^	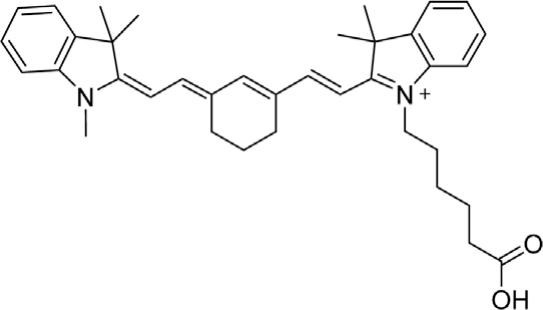	Pre-clinical	- IgG-Cy7 (intradermal delivery for SLN mapping)
				- COC183B2-Cy7 (intravenous delivery for metastatic LN detection)
				- LMWH-NLips/Cy7 (subcutaneous delivery for metastatic LN detection)
				- ND-PG-Cy7 (intravenous delivery for metastatic LN detection)
Cy7.5	MW = 834 Da λ_abs/em_ = 780/810 *ϕ* = 28%	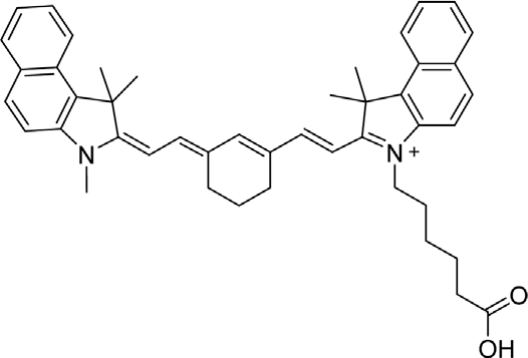	Pre-clinical	- Cy7.5-PEG (intradermal delivery for dermal lymphatic function imaging)
				- TAPA-Cy7.5 (subcutaneous delivery for lymphatic drug delivery to the brain)
Alexa Fluor 680	MW = 857 Da *λ*_abs/em_ = 679/702 *ϕ* = 0.36	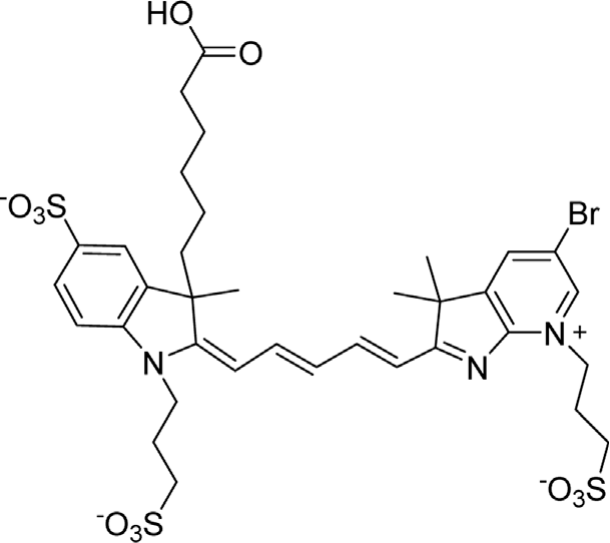	Pre-clinical	AF680-BSA (intradermal delivery for albumin clearance/lymphatic function)
				AF680-BBN (intravenous delivery for detection of prostate cancer metastatic LNs)
IRDye800CW	MW = 962 Da λ_abs/em_ = 774/789 *ϕ* = 12% (serum)	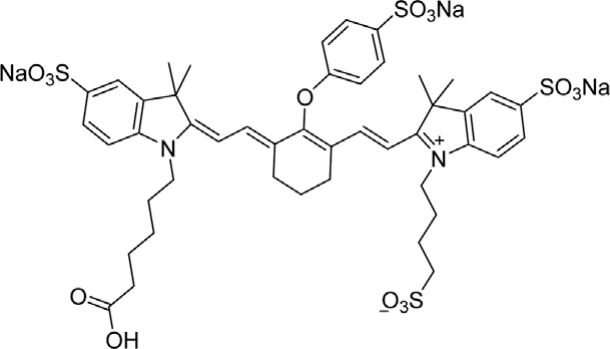	Phase II (for panitumumab-IRDye800)	- HSA-IRDye800 (peritoneal delivery for anatomical studies of peritoneal lymphatics in mice)
				- IRDye800CW-PEG (intradermal delivery for FL)
				- cABD-IRDye800 (intradermal delivery for SLN mapping)
				- Polymer nanogels (intradermal delivery for SLN mapping)
				- Panitumumab-IRDye800/Timanocept-IRDye800 (intravenous delivery for metastatic LN detection)
CH1055 (a D-A-D dye)	MW = 970 Da λ_abs/em_ = 808/1,055 *ϕ* = 0.3%	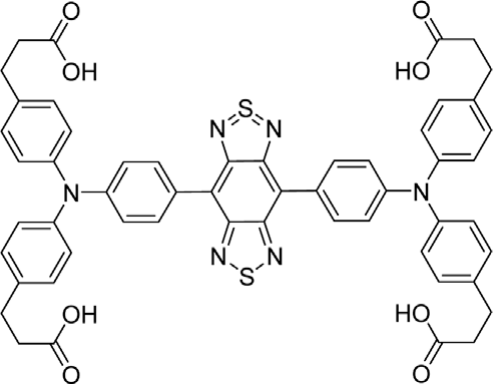	Pre-clinical	- CH1055-PEG (intradermal delivery for NIR-II FL/SLN mapping)
				- CH-4T (intradermal delivery for NIR-II FL/SLN mapping)
BTC1070 (a polymethine cyanine)	MW = 811 Da λ_abs/em_ = 1,014/1,070 *ϕ* = 0.016% (PBS) *ε* = 115,000 M^−1^ cm^−1^	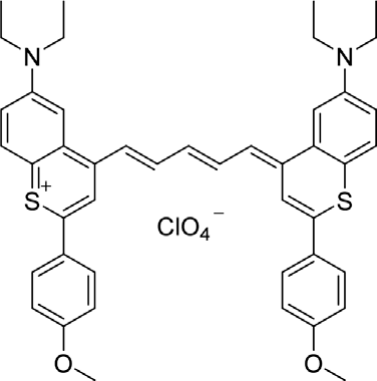	Pre-clinical	- BTC1070 (intradermal delivery for NIR-II FL/SLN mapping)
Single-walled carbon nanotubes (SWCNTs)	Diameter 0.4–1.4 nm Length 1–300 *μ*m λ_em_ = ∼1,000–1,800 *ϕ* = 0.4%	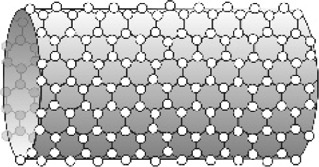	Pre-clinical	- PEGylated SWCNTs (+/- oxygen-doped) (intradermal delivery for NIR-II FL/SLN mapping)
Rare-earth-doped nanoparticles (RENPs)	Diameter ∼10 nm λ_em_ = ∼1,000–1,600 *ϕ* = 20–90% (depending on rare earth ions)	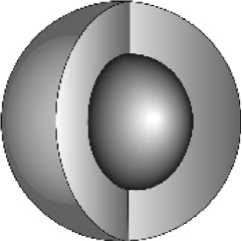	Pre-clinical	- RENPs@Lips (intradermal delivery for NIR-II FL/SLN mapping)
				- DSPE-5KPEG RENPs (subcutaneous delivery for multicolor NIR-II FL/SLN mapping)
Quantum dots (Qdots)	Diameter ∼5–10 nm λ_em_ = ∼500–1,500 *ϕ* = 8–17%	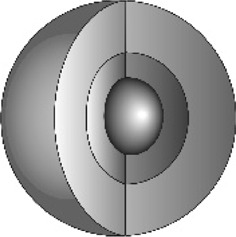	Pre-clinical	- Carboxyl-coated Cd/Se Qdots (intradermal delivery for visible wavelength SLN mapping)
				- Polymer-coated PbS/CdS Qdots (intradermal delivery for NIR-II SLN mapping)

It should be noted that tracer characteristics vary with manufacturer and external conditions. The values given are representative values from the literature. Examples of conjugate tracers are the examples given in this manuscript for lymphatic imaging and are not exhaustive.

AF, Alexa Fluor; BBN, bombesin; BSA, bovine serum albumin; C dots, Cornell dots; cABD, cyclic albumin-binding domain; CLIO, cross-linked iron oxide; CT, computerised tomography; Cy, cyanine; dext, dextran; D-A-D, donor–acceptor–donor; FL, fluorescent lymphangiography; HSA, human serum albumin; IgG, immunoglobulin G; LMWH, low molecular weight heparin; LN, lymph node; mAb, monoclonal antibody; MRI, magnetic resonance imaging; NA, not applicable; ND-PG, polyglycerol-functionalized nanodiamonds; NHS, N-hydroxysuccinimide; NIR, near-infrared; PEG, polyethylene glycol; PET, positron emission tomography; PNG, polymer nanogel; SLN, sentinel lymph node; TAPA, tannic acid-polyvinyl alcohol.

FITC-dextran has been used for fluorescence microlymphography (FML), a technique for visualizing human superficial skin lymphatics pioneered by Bollinger and colleagues four decades ago (Bollinger et al., 1981). In this technique, minute amounts (0.01–0.05 ml of 250 mg/ml solution) of FITC-dextran were delivered into the subepidermal layer *via* a microneedle and the superficial lymphatics were visualized with a fluorescent microscope (Bollinger and Amann-Vesti, 2007). Extension of the fluorescence past 12 mm from the depot strongly supported the diagnosis of lymphedema as it suggested poor clearance into the deeper lymphatics (Mellor et al., 2000; Bollinger and Amann-Vesti, 2007). The superficial lymphatics also showed characteristic morphological abnormalities in various conditions (e.g., microaneurysms in lipoedema), and the dye sometimes reappeared at more proximal superficial sites due to dermal backflow in patients with lymphedema (Bollinger and Amann-Vesti, 2007). The limit of visible light penetration and tissue scattering only allowed one to visualize lymphatics in the stratum papillary layer, but not in the deeper dermal lymphatics (depth limit of 100–150 μm) (Bollinger et al., 1981; Sevick-Muraca et al., 2014). For this technique, Bollinger used 150 kDa FITC-dextran as he found that the superficial dermal lymphatics were permeable to the smaller 40 kDa FITC-dextran (Bollinger, 1993; Fischer et al., 1997). Others have also used 150 kDa FITC-dextran for FML (Stanton et al., 1997; Mellor et al., 2000; Keo et al., 2015). Molecules of this size are likely to perfuse poorly through the interstitium, as mentioned earlier, but presumably because of the small scale involved, this is unimportant for this technique.

**FIGURE 3:**
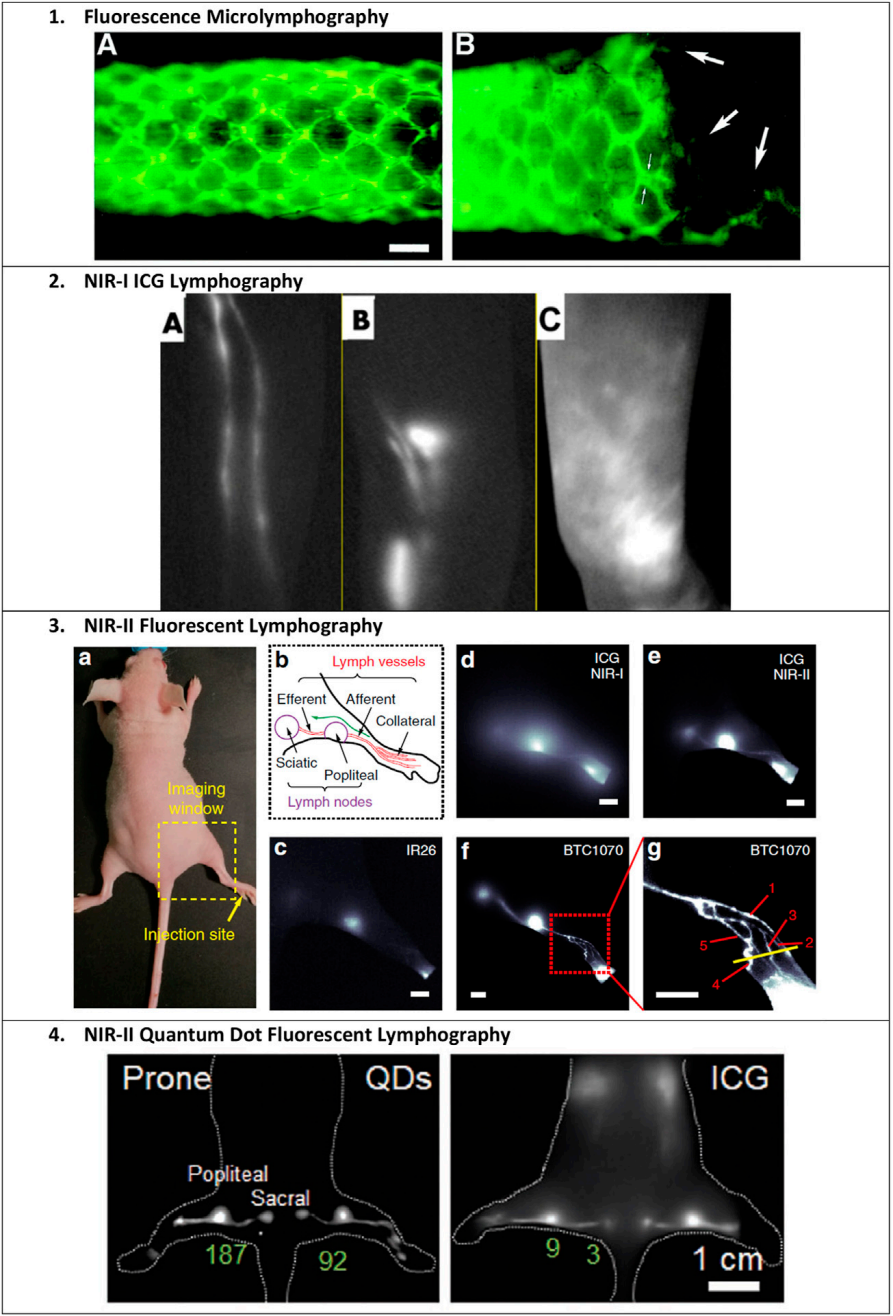
Examples of fluorescent lymphatic imaging. 1) Fluorescence microlymphography (FML) of the nude mouse tail after the injection of 5 μL of 25% 2,000 kDa FITC-dextran at the distal tip. The direction of lymph flow is from left to right. Bar, 400 μm. (A) Typical continuous lymphatic network 22 min after injection. (B) Same tail, 28 days after injection of an FSaII cell suspension. Large arrows indicate attenuated vessels possibly inside the tumor. Small arrows indicate the increased apparent diameter due to engorgement and/or flattening of the lymphatic capillary. Reproduced with permission from Leu et al.(2000). 2) ICG lymphography. Left-arm of a 38-year-old woman with left breast cancer who underwent a mastectomy and axillary lymph node dissection. (A) Before surgery with no edema. ICG lymphography showed a linear pattern. (B) Three months after surgery. Although the patient complained of a heavy feeling in the left upper arm, significant limb volume change was not seen. A splash pattern was observed on the left upper arm. (C) Twelve months after surgery. The left arm became significantly larger clinically despite the use of a compression sleeve. A stardust pattern was observed throughout the left arm. Reproduced with permission from Akita et al. (2016). 3) NIR-II fluorescent lymphography. Images demonstrate the superior resolution of imaging ICG in the NIR-II range and of BTC-1070 compared to ICG and IR26. (a) Digital photograph of a nude mouse fixed on an imaging plate, showing the injection site (yellow arrow) of contrast agents and the lymphatic drainage imaging window (dash square). (b) Schematic illustration of the anatomical structure of the lymphatic system in the hindlimb of nude mice; the green arrow represents the lymphatic drainage from the paw to the sciatic lymph node. (c–g) Fluorescence images of lymphatic drainage using IR26 (c), ICG (d, e), and BTC1070 (f, g) in the hindlimb of nude mice on an InGaAs camera. (g) High-magnification (×3) image of the ankle (red square in f), showing that at least five collateral lymph vessels were resolved. Scale bar, 2.5 mm IR26 and BTC1070 imaging signals were collected at wavelengths of 1,200–1,700 nm under 1,064 nm excitation. ICG was excited at 808 nm, and images were collected in the NIR-I (850–950 nm) and NIR-II (1,000–1,700 nm) regions, respectively. Reproduced with permission from Wang et al. (2019). 4) NIR-II quantum dot fluorescent lymphography. Mouse footpads were injected with 50 μL ultrabright PbS/CdS core/shell quantum dot solution (100 pmol). Shown is a comparison of popliteal and sacral lymph nodes imaged with QDots at the NIR-IIb window and ICG at the NIR-I window, respectively. The signal intensity of LNs is labeled with values in green. Reproduced with permission from Tian et al. (2020).

FML has lacked widespread clinical adaption because FITC-dextran is not officially approved for clinical use despite being well tolerated (Polomska and Proulx, 2021). Furthermore, advances in NIR imaging are now allowing deeper and superior imaging of skin lymphatics over much larger areas. However, it still finds widespread application in animal models. The technique was adapted for the mouse tail, where the flow was measured by the velocity of the fluorescent front as it spread through the honeycomb-like layer of superficial dermal lymphatics (Leu et al., 1994; Leu et al., 2000) (Figure 3). Here the authors used the large 2,000 kDa FITC-dextran, injected intradermally at the tip of the tail. This technique could image lymphatics up to 450 μm deep using standard epifluorescence microscopy (Padera et al., 2002b).

FITC-dextran is also an ideal fluorophore conjugate for surgically exposed lymphatic vessels where the vessel lies superficially in the surgical plane and there is no skin barrier. Liao et al. (2014) developed a method of measuring popliteal lymphatic contractile function in mice using epifluorescence microscopy. Here, 2,000 kDa FITC-dextran (or rhodamine-dextran) was injected subcutaneously into the footpad, and the popliteal lymphatic was imaged after surgical exposure. Similarly, forefoot intradermal injection of 2,000 kDa FITC-dextran was used to assess lymphatic vessel reconstitution in the surgically exposed axilla of mice following LN removal (Tammela et al., 2007).

### 2.2 Microspheres

FITC can be incorporated into microspheres for lymphatic uptake and imaging. Moriondo et al. (2013) used commercially available FITC-conjugated 100 nm polystyrene microspheres, injected into the peritoneal cavity, to visualize diaphragmatic lymphatics in rats. Because of their size, the microspheres could only enter the diaphragmatic lymphatic stomata. They also have the added advantage of less photobleaching as the FITC molecule is impregnated throughout the microsphere.

### 2.3 Enteral Delivery

BODIPY dyes are structurally derived from cyanine dyes; their core structure contains a boron-carbon heterocyclic ring between two pyrrole rings, giving many potential functional modification sites (Table 2) (Wu et al., 2022). This structure increases its molecular rigidity compared to cyanine dyes, leading to excellent brightness, quantum yield, and photostability (Wu et al., 2022). Kassis et al. (2012) used the green fluorescent fatty acid BODIPY® FL C16 (4,4-difluoro-5,7-dimethyl-4-bora-3a,4a-diaza-s-indacene-3-hexadecanoic acid) (ex/em 490/520 nm) to visualize mesenteric lymphatics in rats. This method has the added advantage of being able to measure functional lipid uptake and transport *via* fluorescence intensity (Kassis et al., 2012; Rehal and von der Weid, 2017). Small fatty acids such as BODIPY® FL C16 (474 Da) are easily absorbed into enterocytes and then incorporated into lipoproteins within the enterocyte for delivery to lymphatics (Figure 2B) (Sevick-Muraca et al., 2014; Trevaskis et al., 2015). However, because it is a small molecule, any that is not incorporated into a chylomicron will preferentially be taken up by the blood circulation, as the rate of portal blood flow is 500 times that of mesenteric lymphatic flow (Trevaskis et al., 2015). Therefore, Kassis et al. (2012) delivered it mixed with olive oil to improve lipoprotein incorporation. As mesenteric lymphatics drain into the thoracic duct, BODIPY® FL C16 can also be used for imaging the thoracic duct after surgical exposure (Harvey et al., 2005) and gives a clearer signal than intradermal administration of ICG into the lower limb (Sevick-Muraca et al., 2014).

### 2.4 Lymphatic Vessel and Immune Cell Labeling

Visible fluorescent imaging of lymphatic vessels can also be achieved by using fluorescently-labeled antibodies or cells that home to lymphatic vessels. McElroy et al. (2009) conjugated monoclonal anti-mouse LYVE-1 antibody, which binds to the lymphatic endothelial cell-specific LYVE-1 (lymphatic vessel endothelial hyaluronan receptor 1), with AlexaFluor 488 dye (ex/em 495/519 nm). The authors showed that injection of the AlexaFluor 488-LYVE-1 conjugate into the exposed inguinal LN successfully stained the draining lymphatic vessel for up to 48 h, which was visualized in real-time after surgical exposure. Laakkonen et al. (2002) identified a cyclic 9-amino-acid peptide, LyP1, that homes to lymphatic endothelial cells. They labeled it with fluorescein and found that it co-localized with tumor lymphatics but not blood vessels. Lymphatic vessels can also be fluorescently labeled by labeling the immune cells that are known to drain *via* the lymphatic route. Li et al. (2013) visualized immune cell trafficking (e.g., macrophages, granulocytes, B cells) within popliteal afferent lymphatics by injecting FITC-conjugated anti-CD11b, anti-Gr-1, anti-CD19, anti-CD3, or anti-CD45.2 antibody into mouse footpads, which labeled the draining immune cells, in conjunction with Texas red-conjugated dextran beads to highlight the lymphatic vessel itself.

## 3 Visible Wavelength Fluorophores: Red

Fluorophores that fluoresce red allow slightly deeper visualization compared to green fluorophores and have therefore proved popular for a range of lymphatic imaging techniques. The depth of imaging is still less than NIR fluorophores but does not require a specialist NIR imaging device.

### 3.1 TRITC-Dextran

Rhodamine is another xanthene-derived fluorophore with a structure very similar to fluorescein, but that emits at longer wavelengths (Wu et al., 2022). Tetramethylrhodamine isothiocyanate (TRITC)-dextran (a rhodamine derivative, ex/em 544/572 nm) can also be used for FML (Veikkola et al., 2001). The depth of imaging using TRITC-dextran can again be improved by multiphoton laser scanning microscopy (MPLSM), which has been used, for example, to visualize lymphatics surrounding a superficial tumor in mice (Padera et al., 2002a). MPLSM is a powerful technique that utilizes simultaneous excitation of the fluorophore (FITC or TRITC) by two photons at a less energetic infrared wavelength (780 nm) (Padera et al., 2002b). Because of the requirement for simultaneous excitation by two photons, excitation only occurs at the focal point of the objective, giving higher spatial resolution for a greater depth of imaging (Padera et al., 2002b). This technique allows visualization of the deeper collecting lymphatics of the skin (Padera et al., 2002b).

### 3.2 Cyanine Dyes: Cy5 and Cy5.5

#### 3.2.1 Introduction to Cyanine Dyes

Cyanine dyes, spanning both visible and NIR wavelength ranges, are important widely-used fluorophores for lymphatic imaging and include the most commonly used fluorophore for lymphatic imaging, indocyanine green (discussed in Section 4). They were first discovered by English chemist Charles Hanson Greville Williams over a century ago and since then, this important class of organic functional dyes has grown exponentially and been used in a wide range of biomedical applications.

**FIGURE 4:**
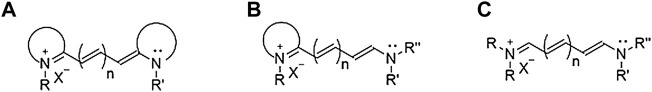
General structures of **(A)** cyanines, **(B)** hemicyanines, and **(C)** streptocyanines.

Cyanine dyes are characterized by a conjugated chain of methine (–CH=) units linking two heterocyclic rings containing nitrogen centers, one of which is positively charged (Figure 4) (Gopika et al., 2021). They can be classified according to the number of methine groups, e.g., pentamethine cyanine (five methine units, *n* = 2 in Figure 4) (Gopika et al., 2021). They can also be structurally defined based on the terminal groups in the structure as cyanines (contain a heterocyclic moiety at both ends), hemicyanines (contain a heterocyclic moiety at one end only), and streptocyanines (contain a non-cyclic group at both ends) (Figure 4) (Ilina and Henary, 2021).

The synthesis of novel cyanine dyes is of interest because of their exceptional physicochemical and optical properties, including high fluorescence brightness, high pH sensitivity, excellent molar absorption coefficients, high photochemical stabilities, and narrow absorption bands (Ilina and Henary, 2021). Over the past few decades, several cyanine dyes have been synthesized through modification of the heterocyclic moiety or polymethine chain to obtain a new generation of cyanine dyes with improved characteristics such as improved photostability, decreased phototoxicity, increased fluorescence brightness, and improved aqueous solubility (Gopika et al., 2021).

Important red cyanine dyes used in lymphatic imaging include Cy5 (ex/em ∼649/666 nm) and Cy5.5 (ex/em ∼675/694 nm) (Table 2). Techniques for targeting these fluorophores to lymphatics include, among others, incorporation into fatty acid nanoemulsions for enteral delivery, lymphatic-targeted fluorescently-labeled antibodies or cells, enzymatic activation at the LN, or incorporation into larger carriers such as dendrimers.

#### 3.2.2 Enteral Delivery

Like BODIPY® FL C16, Cy5 can be conjugated to a fatty acid for enteral delivery and subsequent uptake by mesenteric lymphatics. In an attempt to increase delivery of the GLP-1 receptor agonist exenatide to the pancreas, Lin et al. developed a lipid-based oil-structured nanoemulsion (NEM) system for enteral delivery and subsequent mesenteric lymphatic transport of the drug to the pancreas in type 2 diabetic rats (Lin et al., 2019a). The medium-chain (C10) fatty acid capric acid was used as the basis for the NEM. Although medium-chain fatty acids are traditionally thought to predominantly enter the portal venous circulation (Babayan, 1987), capric acid is sufficiently large enough to enter lymphatics (Ikeda et al., 1991). To check the biodistribution of the NEM system in rats, the authors labeled the drug-NEM with Cy5-N-hydroxysuccinimide (NHS) and the vehicle-NEM with DiO (3,3′-dioctadecyloxacarbocyanine perchlorate; ex/em 484/501 nm) and found that both had accumulated in the pancreas after 5 h. They confirmed that the transport between the intestine and the pancreas was *via* lymphatics by using cycloheximide, which is known to inhibit chylomicron secretion from the enterocytes in villi and the phagocytic activity of M cells in Peyer’s patches, thereby preventing lymphatic transport of fatty acids from the intestine (Attili-Qadri et al., 2013). Successful fluorescence was detected in the pancreas following duodenal administration of the conjugates, showing that passive pancreatic targeting could be achieved through oral administration and subsequent mesenteric lymphatic uptake.

#### 3.2.3 Cornell Dots

Cornell dots are small silica spheres (<8 nm diameter) that provide a chemically inert encasement for a variety of fluorescent dyes. For *in vivo* use, they are coated with PEG to prevent immune activation. Zanoni et al. (2021) tested the accuracy of the intradermal administration of cRGDY-PEG-Cy5.5-nanoparticles (Cy5.5 Cornell dots surface modified with PEG and cyclic arginine-glycine-aspartic acid-tyrosine peptides) for SLN mapping in patients with head and neck melanoma. The cRGDY peptide conjugates targeted the particles to a_V_ integrins, which are highly expressed on neoangiogenic endothelial and melanoma cells. The Cornell dots were safe at the microdoses given (2 nmol in 0.4 ml) and still showed a very high maximum SBR of 40 for the SLN.

#### 3.2.4 Immune Cell and Cancer Cell Labeling

Red fluorescent cyanine dyes can also be targeted to lymphatic vessels and nodes by utilizing immune and cancer cell targets. Prior to the widespread clinical use of ICG, minimally invasive SLN mapping was demonstrated by conjugating Cy5 to vitamin B_12_. McGreevy et al. (2003) demonstrated that intradermal injection of Cy5-B_12_ into healthy pig hindlimbs allowed laparoscopic visualization of inguinal LNs after laser excitation. In this study, the authors showed that SLN detection using Cy5-B_12_ was possible in healthy tissue but the additional benefit of using B_12_ is that it accumulates in rapidly dividing cells such as tumor cells, thereby having the potential to identify metastatic LNs in real-time (McGreevy et al., 2003).

Cyanine dyes can also be conjugated to antibodies targeting immune cells that home to lymphatic vessels. Cy5.5-NHS has been conjugated to rituximab (Cy5.5-Rit, ∼150 kDa), a clinically approved chimeric monoclonal antibody that targets the B-cell marker CD-20 (Li et al., 2016). In mice, Cy5.5-Rit fluorescence was detected in the axillary SLN 30 min after subcutaneous injection into the forefoot and was detectable up to 9 days post-injection (Li et al., 2016). Cy5.5-Rit has also been used to detect CD-20^+^ lymphoma cells, and thereby lymphoma-containing LNs, reaching the LNs *via* the blood after intravenous or intraperitoneal delivery (Biffi et al., 2008).

Non-specific mouse polyclonal IgG has also been used for lymphatic targeting of red cyanine fluorophores because it is rapidly taken up by lymphatics, shows low vascular permeability, and has a good toxicity profile (Hama et al., 2007). Hama et al. (2007) demonstrated two-color SLN mapping in mice by simultaneous intradermal injection of Cy5.5-IgG and the NIR Cy7-IgG into various different sites (forefoot, breast, hindfoot, etc.), followed by imaging of the respective draining LNs. To separate the colors, the authors performed spectral imaging (Maestro *In Vivo* Imaging System, CRI Inc., Woburn, MA), which incorporated spectral unmixing algorithms. SLNs were identified as early as 1 minute post-injection, and they remained detectable for 90 min. Penetration was about 5 mm. Use of this method can allow one to spare unaffected LNs and therefore reduce the risk of post-operative lymphedema.

#### 3.2.5 Lymph Node Enzymatic Activation

Wunderbaldinger et al. (2003) tested an enzyme-activated fluorescent macromolecular probe called C-PGC, consisting of a poly-L-lysine backbone with attached Cy5.5 molecules, sterically protected by multiple methoxyPEG side chains (450–500 kDa). This copolymer has previously been used in pre-clinical trials as a vehicle to deliver drug therapies to lymphatic nodes (Callahan et al., 1998). C-PGC also contains 44 unmodified lysines that can be enzymatically cleaved by cathepsin B (Weissleder et al., 1999), which is commonly found in LNs (Crocker et al., 1984). Before enzymatic cleavage, the Cy5.5 molecules are quenched due to their overlapping excitation and emission spectrums, but after enzymatic cleavage, Cy5.5 molecules are separated and the fluorescent signal increases by 30-fold (Wunderbaldinger et al., 2003). The authors showed clear fluorescence of LNs around the body after intravenous administration and draining axillary and clavicular LNs after subcutaneous injection into the forefoot (Wunderbaldinger et al., 2003). Fluorescent tracers can be joined to MRI contrast agents to combine the high sensitivity of the former with the high spatial resolution of the latter (Talanov et al., 2006). Cy5.5-PGC has also been synthesized to include cross-linked iron oxide amine (CLIO) and both MRI and fluorescence LN imaging after a subcutaneous injection has been demonstrated in mice (Josephson et al., 2002).

#### 3.2.6 Dendrimers

Dendrimers are symmetrical polymeric molecules that are useful for lymphatic targeting due to their tunable well-defined sizes and large number of accessible functional groups. The biocompatible and water-soluble dendrimer polyamidoamine (PAMAM) has gained particular attention for bioimaging, including lymphatic imaging, as its internal architecture closely mimics proteins and peptides (Nune et al., 2011). Talanov et al. (2006) were the first to show that these can be used as a platform for another fluorescent-MRI dual imaging agent with Cy5.5 and gadolinium (III). Here, the authors showed accumulation in the axillary LN after injection in the mammary fat pad, with lymphatic targeting enhanced by the size of the molecule. This latter study reveals how Cy5.5 can also be considered a NIR molecule, as the fluorescent LN was invisible to the naked eye despite an intense signal in the NIR through the skin (excitation light filtered to 615–665 nm and the multispectral camera set for 720 nm emission spectra). PAMAM-G6 dendrimers have also been used for multicolor SLN mapping in mice after conjugation with Alexa Fluor 660, 700, and 750, and diethylene triamine penta-acetic acid (DTPA) coating (Kobayashi et al., 2009a).

## 4 NIR-I: Indocyanine Green

NIR fluorophores offer the primary advantage over visible wavelength dyes of deeper tissue penetration and therefore are commonly used in non-invasive FL. ICG is an FDA-approved NIR-I fluorophore used clinically in lymphatic imaging (the other FDA-approved NIR fluorophore is methylene blue (ex/em 665/686)). It was originally approved for the measurement of hepatic clearance in the 1950s, and its use in lymphatic imaging remains off-label (Polomska and Proulx, 2021). It is inexpensive, safe (LD_50_ = 50–80 mg/kg), and rapidly cleared by the liver (T_1/2_ in plasma is 3–4 min) (Reinhart et al., 2016). In addition to lymphography, it has been used extensively in various clinical applications, including cardiology, neurosurgery, and ophthalmology (Reinhart et al., 2016). ICG has certain characteristics, including its excellent safety profile (especially for an imaging contrast agent), that makes it a highly valuable fluorophore for lymphatic imaging. It is a negatively charged tricarbocyanine dye possessing both hydrophilic and lipophilic properties (Table 2). While it is water-soluble (1 mg/ml), it is relatively hydrophobic and has a pKa of 3.27 (Schaafsma et al., 2011; Alander et al., 2012). Although it has a low molecular weight (751.4 Da) and is relativity inert (Miwa, 2016; Reinhart et al., 2016), *in vivo* it binds extensively to proteins, notably albumin and high- and low-density lipoproteins, which increases its effective size, thereby decreasing rapid movement through the interstitium and preventing ICG aggregation (Reinhart et al., 2016). It is most likely that, because of the low molecular weight of ICG, protein binding assists lymphatic uptake (although, as detailed as follows, there is little evidence that pre-mixing with albumin assists lymphatic uptake).

**FIGURE 5:**
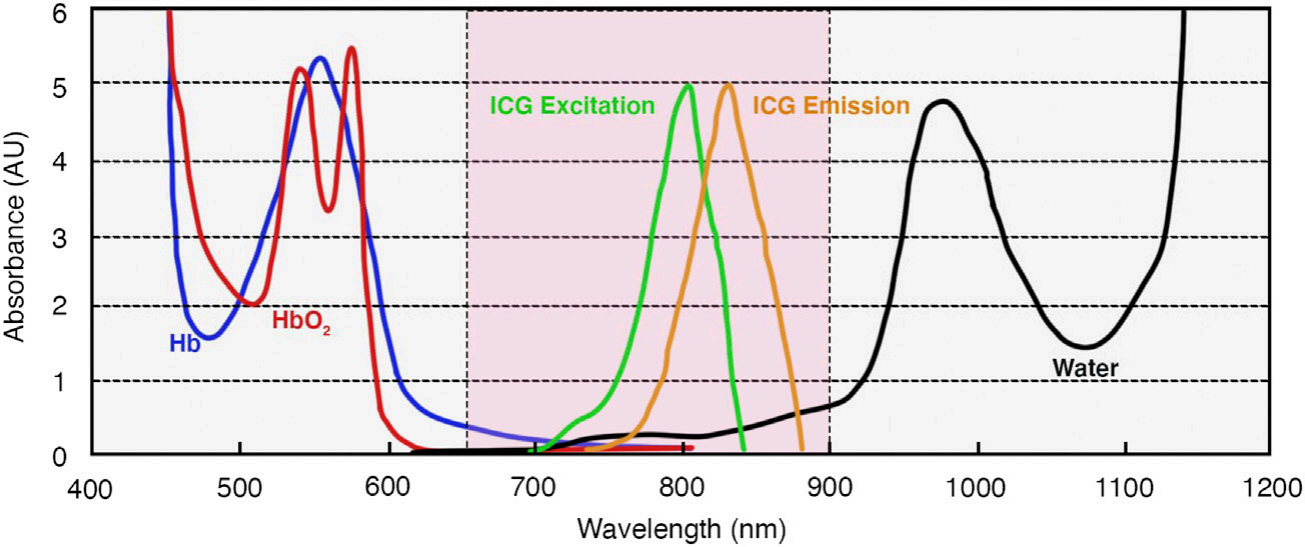
Optical characteristics of ICG and absorption spectra of hemoglobin and water. Based on information from Miwa (2016). The area in pink represents the “optical window” (650–900 nm) of the far-red/NIR-I spectrum for optimum *in vivo* imaging. Abbreviations: AUs, absorbance units; ICG, indocyanine green; Hb, deoxygenated hemoglobin; HbO_2_, oxygenated hemoglobin.

The excitation and emission of ICG is 750–800 nm and 820–840 nm, respectively (Reinhart et al., 2016). Therefore, it falls within the “optical window” of *in vivo* imaging (Figure 5), giving it high contrast and a depth penetration that is ideal for non-invasive lymphography (Miwa, 2016). The peak excitation and emission are dependent on formulation and interaction with other molecules in their suspended state. The excitation peak shifts from 780 nm in an aqueous solution to 805 nm when bound to albumin (Mordon et al., 1998), and the emission peak shifts from 808 nm to around 820 nm (Polomska et al., 2019). In plasma, the absorption and emission peaks of ICG are around 807 and 822 nm (Schaafsma et al., 2011). Furthermore, the fluorescent intensity increases when ICG is mixed with fetal bovine serum (FBS) or human serum albumin (HSA) (He et al., 2020), and the quantum yield has been shown to increase by nearly 3.5 times when it is mixed with FBS (Jung et al., 2013). Mordon et al. (1998) suggested that, in plasma, some ICG binds to blood endothelial cells, further increasing the maximum absorbance wavelength from 826 to 835 nm, but it is unknown if the same process occurs in lymphatic vessels.

ICG fluorescence is usually captured by charge-coupled device (CCD) image sensors, or more recently the less expensive complementary metal-oxide semiconductors (CMOS), which are active-pixel sensors commonly found in digital cameras (DSouza et al., 2016; Miwa, 2016) [for reviews on NIR imaging devices see Zhu and Sevick-Muraca (2014) and DSouza et al. (2016)]. Like all NIR fluorophores, the imaging system must be matched to the Stokes shift (difference in the peak excitation and emission). ICG has a relatively narrow Stokes shift and therefore the imaging filters used are of particular importance (DSouza et al., 2016). The choice of filter depends on the imaging system and excitation source, taking into account the *in vivo* wavelength shifts noted earlier. A pre-clinical example is 808 nm laser diode excitation with emission captured *via* a 840 nm CW bandpass filter (Weiler et al., 2012). A range of propriety clinical devices are also available (Zhu and Sevick-Muraca, 2014; DSouza et al., 2016).

### 4.1 Applications of ICG

FL in humans predominantly makes use of ICG, which was re-purposed in the mid-2000s by Kitai et al. (2005) and Sevick-Muraca et al. (2008) for detecting SLNs in breast cancer patients, Ogata et al. (2007) for surgical guidance of lymphaticovenular anastomoses, and Unno et al. (2007) for investigation of lower limb secondary lymphedema. For lymphatic imaging, it is most often injected intradermally, where draining lymphatic vessels and nodes can then be detected up to 3–4 cm below the skin using intensified imaging devices (Sharma et al., 2007; Sevick-Muraca et al., 2008; Sevick-Muraca et al., 2014). Spatial resolution depends on the imaging device but also declines with increasing depth, with the maximum depth for high resolution reported to only be around 3 mm (Teng et al., 2021). Other common administration routes include peri-tumor and subcutaneous injection, often aided by external mechanical compression (Szuba and Rockson, 1997). The main indications for ICG lymphography are staging and microsurgical treatment of secondary lymphedema and SLN mapping in melanoma and breast cancer (Burnier et al., 2017). There have been over 50 clinical trials showing the numerous benefits of ICG over blue dye and radiocolloids in SLN detection for a range of cancers (Hameed et al., 2019) [including, for example, esophageal (Wang et al., 2021), bladder (Manny and Hemal, 2014), and colon (Andersen et al., 2017; Son et al., 2021)]. Unlike blue dye or radiocolloids, ICG with NIR imaging allows one to visualize draining lymphatics in real-time and more accurately assess lymphatic vascularization around the SLN, allowing better preservation of axillary lymphatics in breast cancer surgery, decreasing the risk of secondary lymphedema (Aoyama et al., 2011; Burnier et al., 2017). This risk can also be assessed by using axillary reverse mapping, where ICG is subcutaneously injected pre-operatively into the interdigital area of the ipsilateral hand (Sakurai et al., 2014). Patients where the arm lymphatics drained into the breast SLN were found to be at increased risk of lymphedema following SLN biopsy (Sakurai et al., 2014). If desired, ICG can be used in conjunction with sulfan blue (Hirano et al., 2012) or patent blue (van der Vorst et al., 2012; Guo et al., 2014) to more clearly see lymphatic vessels in visible light, or ^99m^Tc-nanocolloid (Burnier et al., 2017) to locate the general area of the SLN prior to skin incision. As well as SLN mapping, it has also been used clinically for diagnosis (Mihara et al., 2012), staging (Yamamoto et al., 2011), surgical planning (Hara and Mihara, 2019), and treatment efficacy (Zaleska and Olszewski, 2018) of lymphedema, lymphatic remodeling over time (Rasmussen et al., 2017), detection of lymphatic leaks (Chan et al., 2018), and assessing lymphatic flow after transplant surgery (Yamamoto et al., 2016). It has also been used for human cadaver anatomical studies after intradermal injection and gentle massage to move the dye proximally along the lymphatic vessel (Suami, 2017).

#### 4.1.1 Functional Imaging

Soon after its use in SLN mapping was realized, it became clear that non-invasive FL in humans was able to provide functional as well as structural information. The leading edge of ICG fluorescence along the draining lymphatic can be followed in real-time, only minutes after injection (Kitai et al., 2005). Sevick-Muraca et al. (2008) were the first to also detect the active pumping of lymph in humans using non-invasive ICG FL. They visualized propelled “packets” of lymph along the vessel after peri-areolar and peri-tumor injections in the breast, where lymph packet velocities were 0.08–0.32 cm/s and durations between propulsion events were 14–92 s (Sevick-Muraca et al., 2008). Around the same time, non-invasive imaging of functional lymphatic activity was also demonstrated in the pig abdomen and hind-limb (propulsion of 2–16 cm length at 0.5–3.3 pulses/min and velocities of 0.23–0.75 cm/s, independent of respiration rate) (Sharma et al., 2007) and the mouse (lymph packet velocities were 1.3–3.9 mm/s (tail) and 0.28–1.35 mm/s (axilla), with frequencies of 1.17/min on average (tail) and 0.72–11.1/min (axilla) (Kwon and Sevick-Muraca, 2007), allowing longitudinal functional imaging of both humans and animal models to assess disease progression and treatment effects. The distance over which these packets of lymph are propelled can be much longer than an individual lymphangion (Liao et al., 2014), suggesting coordinated contraction of adjacent lymphangions, which has been demonstrated *in vivo* (Russell et al., 2022) and in *ex vivo* isolated lymphatics (Castorena-Gonzalez et al., 2018).

#### 4.1.2 Formulation and Dose

For most clinical applications, ICG is either suspended in sterile water (followed by dilution with saline if an isotonic solution is needed (Alander et al., 2012)) or pre-mixed with HSA (Boni et al., 2015). As mentioned earlier, lymphatic targeting of ICG occurs because it binds with interstitial proteins, increasing the effective particle size. However, the extent of protein binding is inconsistent (Proulx et al., 2013), varying with different levels of tissue hydration, and some may enter venous capillaries (Proulx et al., 2010). HSA is around 66.5 kDa in weight with an ellipsoid shape of 3.8 × 15 nm (Abdallah et al., 2020). Pre-mixing with albumin is thought to enhance lymphatic uptake and therefore lymphatic fluorescence (Weiler et al., 2012). However, a randomized trial of ICG with or without premixing with 200 mg/ml albumin found no difference in fluorescence brightness of the SLN in 18 patients undergoing breast cancer surgery (Hutteman et al., 2011). In pig lungs, there was no difference between ICG and ICG-albumin in fluorescence intensity in the SLN but there was a 2-min delay in fluorescent detection with the latter (Oh et al., 2013).

ICG-albumin is still a relatively small composite (Mieog et al., 2011), which is beneficial for rapid transport through the interstitium, usually reaching the draining LN within 15 min (Boni et al., 2015). Fujisawa et al. showed that ICG gave a more rapid signal in the draining LN than the traditional ^99m^Tc-stannous colloid radioisotope, but noted that, because of its small size, ICG-albumin is at risk of rapidly spilling into secondary nodes in SLN imaging (Fujisawa et al., 2012). Size has been shown to be important for SLN imaging, with larger molecules generally being retained in the node and producing greater fluorescence (Polomska and Proulx, 2021). There is currently no consensus on the optimal size of the fluorescent tracer for this indication, with suggestions ranging from <50 nm (Li et al., 2015) to 100 nm (Yang et al., 2017).

An increasing concentration of ICG yields higher fluorescence but only within a narrow concentration range. This is because higher concentrations lead to ICG aggregation and quenching, causing a decrease in fluorescence intensity (Miwa, 2016). Aggregation occurs due to van der Waals forces between aromatic regions of ICG molecules (Reinhart et al., 2016), and is prevented by protein binding (Mordon et al., 1998). However, at high ICG concentrations, the capacity of interstitial proteins to prevent aggregation is exceeded (Proulx et al., 2010). The ICG dose reported in the literature is somewhat variable (Hameed et al., 2019), even within the same indication, but is usually kept below 0.5–1 ml at 5 mg/ml in humans, which is where quenching has been observed (Kitai et al., 2005). Sevick-Muraca et al. (2008) showed that successful axillary SLN detection is possible in humans after intradermal administration of only 10 μg ICG in 0.1–0.3 ml volumes.

### 4.2. Novel ICG Formulations

ICG has proven to be a very useful fluorophore for both clinical and non-clinical applications. However, despite its success, it still has disadvantages and limitations that leave room for further refinement. First, its quantum yield is relatively low (0.02 at ex/em 780/830 nm (Sevick-Muraca et al., 1997), compared to Cy5.5 with a quantum yield of 0.2 (Mujumdar et al., 1993)), although this has been shown to improve with protein binding (0.093 in serum) (Moody et al., 1999; Hong et al., 2017). The change in quantum yield with protein binding is also problematic for quantitative studies of fluorescence, where the extent of protein binding is unknown (Babity et al., 2020). Its tendency towards aggregation and self-quenching also means that it is unstable in solution and must be used within hours (Saxena et al., 2003; Saxena et al., 2004), which again can be improved somewhat by pre-mixing with albumin (Zhu and Sevick-Muraca, 2014). In solution, it is also vulnerable to photodegradation and increased degradation at higher temperatures (Saxena et al., 2003) but is more stable in plasma and lymph (Holzer et al., 1998). Other limitations include the relatively small size (leading to the potential for rapid transit through the SLN or drainage *via* blood vessels) (Proulx et al., 2013), poor hydrophilicity, and lack of a functional group for conjugation to adjunct molecules (Rasmussen et al., 2009). ICG itself can also impair lymphatic contractile function (Gashev et al., 2010; Weiler and Dixon, 2013). Finally, ICG is not suitable for patients with an iodide allergy (Alander et al., 2012). These limitations have led researchers to seek both novel formulations of ICG and other NIR fluorophores that can be developed for human use. Novel ICG formulations will now be considered.

#### 4.2.1 Charge

Conjugation of ICG with positively charged colloidal agents can not only stabilize it but also facilitate uptake into the lymphatic vasculature. Lee et al. showed that changing the surface charge on a ICG nanoemulsion from negative to positive by incorporating the cationic lipid stearyl amine (SA) increased lymphatic uptake after injection in mouse footpads and increased the stability of the ICG complex, preventing degradation as well as aggregation (Lee et al., 2015). The authors attributed this to the electrostatic interaction between anionic ICG and cationic SA.

#### 4.2.2 ICG-Loaded Liposomes

Incorporating ICG into liposomes not only improves the stability in solution and *in vivo*, but also increases the size, making uptake into blood capillaries less likely. Proulx et al. (2010) studied the effects of incorporating ICG into 1,2-oleoyl-*sn*-glycero-3-phosphocholine (DOPC) and 1,2-distearoyl-*sn*-glycero-3-phosphoethanolamine-N-[methoxy(polyethylene glycol)-2000] (DSPE-mPEG)-based liposomes (diameter ∼60 nm). They reported that liposomal ICG (LP-ICG), injected intradermally in mice, showed increased fluorescence of draining lymphatics and nodes with a wavelength shift towards longer wavelengths (λ_em_ = 810–831 nm), improving the SBR and depth of imaging. In addition, stability in the solution was improved and excretion in the bile was slowed. Photostability was also improved; LP-ICG maintained its spectral properties for around 14 days, while free ICG had completely lost fluorescence at 7 days. Surprisingly, despite the increased size, the authors found no differences between LP-ICG and ICG in time to reach and flow through the SLN, but by comparing the time to peak enhancement of fluorescence in the SNL compared to the liver, LP-ICG showed less uptake into the blood than free ICG. The authors concluded the free ICG must have entered the blood either *via* venous capillaries at the injection site or *via* high endothelial venules in the LNs.

Surface properties of the liposome are more important than size in determining retention within the LN. Steric stabilization of liposomes by coating their surfaces with 20 nm hydrophilic polyethylene glycol (PEG), forming 100 nm PEG-liposome complexes, avoided macrophage phagocytosis and increased retention within the LN (Oussoren et al., 1997; Oussoren and Storm, 2001). Kraft and Ho also found higher emission intensity, *in vivo* stability, and image resolution of draining lymphatics after subcutaneous injection of LP-ICG coated with DSPE-mPEG2000 (optimal lipid: ICG molar ratio of 250:1) in mice, with stability up to 8 months in the dark at 4°C (Kraft and Ho, 2014). Free ICG in buffer presented 0.3 ± 0.2% of its initial fluorescence, while LP-ICG measured around 78.2 ± 2.8% of its initial fluorescence. This increased photostability aids in clinical settings where intense light can be a challenge. These authors also showed that LP-ICG decreased lymphatic vessel leak and increased the quantum yield, giving markedly improved SBR and spatial resolution (0.2 mm and 5–10 cell resolution) of lymphatic vessels (Kraft et al., 2018).

#### 4.2.3 ICG-Loaded Micelles

Some characteristics of ICG can also be improved by encapsulation in micelles (single-layer aggregates of surfactant phospholipid molecules). Kolliphor HS15 (polyoxyl 15 hydroxystearate) is a nonionic solubilizer and emulsifying agent that has been used to generate ICG-micelles (ratio ∼ 2,000:1 Kolliphor HS15:ICG) (Meyer et al., 2014). ICG-Kolliphor HS15 has a diameter of 13 nm and ex/em of 798/825 nm (Meyer et al., 2014). It remained fluorescent for up to 30 days (compared to 1 day for free ICG) and had low toxicity (Meyer et al., 2014). Polomska et al. showed that relative to free ICG, ICG-Kolliphor HS15 increases fluorescence intensity by 20-fold, which is another 4-fold increase on liposomal ICG (Proulx et al., 2010; Polomska et al., 2019). As well as increasing the size of ICG to improve lymphatic uptake, Kolliphor HS15 also prevented self-quenching, increased the quantum yield, and improved the dye stability in solution (Polomska and Proulx, 2021).

#### 4.2.4 Extended Wavelengths

Although ICG has an emission spectra peak in the NIR-I range, it displays a long tail that can be detected in the NIR-II and SWIR ranges using appropriate sensors. The potential benefit of doing this is the lower tissue autofluorescence and scattering, giving improved SBR, contrast, and spatial resolution, and although other NIR-II/SWIR fluorophores exist, ICG is FDA-approved (He et al., 2020; Swamy et al., 2021). Carr et al. (2018) used 808 nm excitation light and an indium gallium arsenide (InGaAs) SWIR sensor (NIRvana 640, Princeton Instruments) with 1,000–1,400 nm long-pass filters to interrogate ICG after subcutaneous injection in mice hind feet and tails. Lymphatic vessels and nodes were successfully imaged in real-time through the skin. They found that image contrast improved at longer wavelengths but deeper signals were attenuated above 1,400 nm. The authors concluded that NIR-I imaging is appropriate for ICG lymphangiography if the image is not contrast limited, but if it is, longer wavelengths may be useful. He et al. visualized mouse lymphatics using a 1,500 nm long-pass filter and InGaAs sensor (He et al., 2020). They found that fluorescent intensity at 1,500–1,700 nm was greatly enhanced by premixing ICG with 4% HSA, compared to 0%, and that the SBR was much higher at this wavelength compared to shorter wavelengths. Successful NIR-II imaging of liposomal ICG has also been demonstrated, but the authors did not visualize lymphatics (Bhavane et al., 2018).

The ICG molecule itself can also be modified to increase its fluorescence at longer wavelengths. Swamy et al. (2021) shifted the absorption and emission peaks by ∼200 nm by synthesizing a two double-bond extended ICG analog, called ICG-C11. Using ICG-C11, the authors showed higher resolution FL images in mice at 1,100 nm emission compared to 900 nm. They further created an NHS ester derivative (ICG-C11-NHS) for molecular probe attachment, in this case, anti-HER2 and anti-EGFR, targeting tumor cells in mice.

## 5 Other NIR-I Fluorophores

Because of its FDA-approved status, NIR FL is dominated by ICG, but other NIR dyes [e.g., Cy7, Cy7.5, AF680, IRDye680, and IRDye800CW (Table 2)] have also been used in lymphatic imaging and have proved promising in a number of pre-clinical studies. Their main advantage over ICG is the ease of conjugating or incorporating them into different macromolecules, altering their pharmacokinetics but not their optical properties and thus making them highly versatile (Lucarelli et al., 2009; Hong et al., 2017). They are also usually brighter than ICG, although less bright than quantum dots (Kobayashi et al., 2007a), are usually positively charged, and often have a high quantum yield (0.28 for Cy7 in PBS) (Hong et al., 2017). Most are rapidly cleared by the kidneys, although there is some suggestion that Cy7 and Cy7.5 persist in the skin (Yu et al., 2015). Like ICG, they are prone to photo-bleaching and have relatively broad emission spectra, requiring spectral unmixing if multicolor imaging is used (Lucarelli et al., 2009). These NIR-I fluorophores are in different stages of development. IRDye800CW in particular has been used in multiple early phase clinical trials with a view to FDA approval (Hong et al., 2017). Like ICG, they are small molecules, and so are often modified (e.g., PEGylation, conjugation with albumin or dextran, or incorporation into nanoparticles or lipid carriers) for targeted delivery to lymphatic vessels (Table 2).

### 5.1 PEGylation

As mentioned earlier, PEGylation increases the effective molecular size of the molecule, making lymphatic uptake more likely, and decreasing immunogenicity. Cy7.5 NHS was conjugated through amine coupling to a 20-kDa PEG polymer (Babity et al., 2020). The resulting Cy7.5-PEG conjugate was incorporated into the tip of dissolving polymeric microneedles. These microneedles (480 μm in length) were then inserted into rat skin to produce a controlled extended-release of Cy7.5-PEG, producing a fluorescent “tattoo.” The dye was located at the tip of the needles to maximize dye delivery to the dermis, rather than the non-draining epidermis. Due to the high specificity of the Cy7.5-PEG for lymphatic drainage, measuring the fluorescence of the tattoo over time allowed for continuous monitoring (up to 24 h) of the dermal lymphatic drainage of the area.

Chong et al., 2016 developed a method of visualizing mouse flank lymphatic contractile function after surgical exposure and LN infusion of P20-D680 (20 kDa mPEG amine with IRDye680LT). Although the lymphatic system was accessed *via* direct LN infusion, PEGylation still reduced lymphatic leak. P20-D680 and its 40 kDa version P40-D680 have also been used for non-invasive determination of collecting lymphatic vessel contractile function in mice's lower limbs after intradermal administration in the foot due to their high specificity for lymphatic uptake (Blum et al., 2014). The main attraction of IRDye680 is its brightness, allowing high spatial resolution (Proulx et al., 2013). Proulx et al. (2013) developed 10, 20, and 40 kDa PEGylated IRDye680LT and IRDye800CW, again for non-invasive lower limb FL in mice, and compared these fluorophores to non-PEGylated IRDye and liposomal ICG. They found that non-PEGylated IRDye800CW (2.2–2.6 nm diameter) predominantly entered blood vessels after intradermal injection and, of that in lymphatics, leaked from collecting lymphatic vessels. The 10 (diameter 6.2 nm), 20 (diameter 8.4 nm), and 40 (diameter 11.6 nm) PEGylated fluorophores showed increasing lymphatic perfusion and retention within the LN with size, which was comparable with liposomal ICG (diameter 67 nm).

A recent study developed a method of simultaneous multichromatic NIR imaging of both venous and lymphatic uptake from the same injection site. Bernard et al. (2021) used the chemically inert dye IRDye800CW to target venous uptake and a 40 kDa PEG-IRDye680RD to target lymphatic uptake. After intradermal co-injection into mice tails and intra-articular co-injection into rat knee joints, the draining vessels were imaged on a custom CCD camera using two filters. The method showed great sensitivity in detecting temporal and spatial differences in drainage pathways.

Although highly valuable for FL, the benefits of PEGylation can be put into perspective when comparing retention in the LN to specific molecular targeting. Chen et al. (2020) conjugated Cy7 NHS to COC183B2, a monoclonal antibody that shows high-affinity binding to ovarian cancer tissue. By targeting cancer cells rather than non-specific lymphatic uptake, the authors showed that COC183B2-Cy7 remained in local LNs for up to 7 days (vs. 4 h for PEG-Cy7).

### 5.2 Conjugation With Albumin or Dextran

Increasing the size of a fluorophore by conjugation to albumin or dextran enhances lymphatic uptake and improves retention in lymphatic vessels and nodes. Alexa fluor 680-bovine albumin (AF680-BSA) can be purchased commercially (Karlsen et al., 2012) and has been widely used to calculate albumin clearance by lymphatics in mouse models, where decreased fluorescence over time gives a functional measure of local lymphatic drainage (Papadakou et al., 2015; Steinskog et al., 2016). IRDye800 has also been conjugated to human serum albumin (HSA-IRDye800) and injected intraperitoneally in rats to identify lymphatic drainage routes from the peritoneum (Parungo et al., 2007). Rather than direct conjugation to albumin, Davies-Venn conjugated IRDye800CW-NHS to a cyclic albumin-binding domain (cABD) peptide that shows a strong affinity for albumin (Davies-Venn et al., 2012). The cABD-IRDye800 conjugate (3.4 kDa) showed 5.6x brightness compared to ICG. It was tested by intradermal injections into the base of mice tails shown to reach the LN more rapidly than ICG (2.5 min vs. 15 min) and was retained in the node for longer than IRDye800. Conjugating Alexa fluor 700 to amine dextran (AF700-dext; 500 kDa) is also useful for lymphatic imaging. This conjugate has been used to study lymphatic drainage from a tumor by injecting AF700-dext intratumorally and then measuring SLN fluorescence at set time points (Rohner et al., 2015).

### 5.3 Nanoparticles

Like ICG, incorporation of other small cyanine fluorophores in liposomes is thought to increase stability and aid in targeting lymphatics and retention within them. With this in mind, Ye et al. (2016) prepared ∼64 nm diameter Cy7-incorporated nanoliposomes (NLips) for lymphatic imaging in mice. They then used this platform to specifically target metastatic LNs, which ICG is unable to do, by surface coating the liposomes with low-molecule weight heparin (LMWH), which gives a negatively charged hydrophilic layer that promotes lymphatic uptake. Metastatic tumor cells in the LN secrete heparanase, which degrades the heparin coating, leading to prolonged retention in the node. They showed that the LMWH-NLips/Cy7 showed a 4.8-fold increase in maximum mean fluorescence and prolonged signal in the SLN after subcutaneous administration in the footpad compared to non-LMWH-coated NLips/Cy7. Nanodiamonds (NDs) are carbon-based nanomaterials that provide a large surface area that can be functionalized with various molecules (Ansari et al., 2016). Yoshino et al. showed that surface coating NDs with polyglycerol (PG), instead of the more-commonly used PEG, increases resistance against entrapment and capture by reticuloendothelial cells, preventing accumulation in the liver and spleen (Yoshino et al., 2019). Although the authors were targeting tumor cells rather than lymphatics and gave the ND-PG-Cy7 intravenously to mice, they still showed good uptake and retention in LNs (reached *via* the bloodstream) compared to the liver and spleen.

Surprisingly, using PEGylated nanoparticles as carriers, brain accumulation of a fluorescent tracer has been demonstrated after subcutaneous injection into the neck. Nie et al. developed sub-50 nm Cy7.5-labeled tannic acid-polyvinyl alcohol (TAPA-Cy7.5) PEGylated nanoparticles loaded with L-DOPA (Nie et al., 2021). NIR fluorescent imaging showed that the nanoparticles successfully accumulated in the brain after subcutaneous injection in the mice's neck, compared to liver and kidney accumulation after intravenous administration. The authors concluded that the nanoparticles accumulated in the brain *via* meningeal lymphatics that connect the cervical LNs and the cerebral spinal fluid (Louveau et al., 2015). However, it remains unclear how retrograde flow from the cervical LNs to the meningeal lymphatics could occur and the authors did not speculate on this.

Nanogels are aqueous dispersions of cross-linked polymeric nanoparticles (Martínez et al., 2017). Noh et al. (2012) conjugated IRDye800 to biodegradable pullulan-cholesterol polymer nanogels. Following intradermal injection in mice and pigs, they showed that the nanogel had superior photostability, fluorescent signal, and retention time in the node compared to free IRDye800. Bagby et al. developed a series of anionic star poly-(6-O-methacryloyl-D-galactose) polymer-NIR dye (MetDGal-IR820) conjugates, where the anionic charge of the polymers was increased by modifying galactose moieties in the star polymers with succinic anhydride (Bagby et al., 2012). They found that increased anionic nature was associated with increased lymphatic uptake and retention in draining LNs in mice after injection in the hind footpad, with half-lives of 2–20 h in the popliteal nodes and 19–114 h in the deeper iliac nodes.

### 5.4 Peptide and Antibody Conjugation

An NHS form of AF680 has been conjugated to bombesin (AF680-BBN), which is a neuropeptide with a binding affinity for SLNs containing prostatic cancer. After intravenous administration, half of the AF680-BBN was cleared from the bloodstream within the hour, 30% remained in circulation for 4 hours, and fluorescence in the primary tumor and LNs was detected for up to 6 h (Cai et al., 2013). The conjugate showed an 89.4% sensitivity and 92.9% specificity for detecting metastatic LNs in a mice model of prostatic cancer. This example of cancer-targeting, rather than lymphatic-targeting, again shows how metastatic LNs can be detected following intravenous administration.

Alexa fluor 700 has been conjugated to anti-CD40 antibodies (AF700-CD40 mAb) to validate intratumoral nanofluidic drug-eluting seeds (Chua et al., 2020). As antibodies are primarily absorbed by the lymphatic system, and the drug was delivered intratumorally, the fluorescence in tumor draining LNs can be used to assess the rate of off-target organ exposure. Many other studies have shown the benefit of conjugating targeted antibodies to NIR dyes. Although most are specifically targeted at cancer cells, they also detect metastatic LNs and can differentiate them from benign LNs. IRDye800CW-NHS has been conjugated to panitumumab, an anti-epithelial growth factor antibody, to detect metastatic LNs in humans with head and neck squamous cell carcinoma after intravenous delivery (Nishio et al., 2019). Metastatic nodes could be identified with high sensitivity (84.6%), specificity (94.0%), negative predictive value (99.3%), and acceptable positive predictive value (36.2%). Another antibody that has been conjugated to IRDye800 is tilmanocept (tilmanocept-IRDye800) (Qin et al., 2015). This molecule was further modified by radiolabeling with gallium-68 and technetium-99m, providing a triple imaging modality (gammagraphy, positron emission tomography (PET)-CT, and fluorescence) (Qin et al., 2015).

## 6 NIR-II Fluorophores

The development of new NIR-II fluorophores is of considerable interest and has been the topic of multiple recent reviews (Li et al., 2020a; Zhang et al., 2021a; Yang et al., 2021; Qi et al., 2022; Wu et al., 2022; Yang and Zhang, 2022). The NIR-II window (1,000–1,700 nm) has been sub-divided into two optimized windows of NIR-IIa (1,300–1,400 nm) and NIR-IIb (1,500–1,700 nm), which avoid the peak absorbance of water at ∼1,450 nm (Li et al., 2020a). At wavelengths above 1,000 nm, tissue autofluorescence and light scattering and absorption are decreased considerably when compared with the NIR-I window (Chen et al., 2016; Bhavane et al., 2018; Kraft et al., 2018; Zhang et al., 2021a). This allows for an increased SBR and greater resolution of deeper structures. Disadvantages include lower quantum yields, poor water solubility, and those that emit light in the NIR-IIb range very often show severe aggregation-caused quenching in solution (Li et al., 2020b). The structure of the molecule plays a significant role in the quantum yield, as rigid structures generally favor radiative decay (giving stronger fluorescence) and flexible molecules favor non-radiative decay (decreasing quantum yield and generating heat) (Liu et al., 2021). Solubility can be improved by the addition of hydrophilic functional groups, PEGylation, positive, negative, or zwitterionic charging, or encapsulation into amphiphilic polymers (Yang and Zhang, 2022). Emission spectra are usually recorded using an InGaAs camera, which is based on semiconductor alloys with narrower band gaps than CCDs or CMOS cameras (Li et al., 2020a). They often require cooling to –20°C (or lower) to minimize thermal noise and increase light-detection sensitivity (Hong et al., 2017). NIR-II fluorophores are broadly divided into organic and inorganic materials. The benefit of inorganic nanomaterials is their photostability and tunable nature, in terms of structure, size, and functional groups, but they often show poorer biocompatibility compared to organic dyes (Qi et al., 2022). Space prohibits a complete review of studies that have investigated NIR-II fluorophores for lymphatic imaging but instead here we will highlight some general strategies for lymphatic imaging using some recent examples. NIR-II quantum dots for lymphatic imaging will be discussed in Section 7.

### 6.1 Organic NIR-II Fluorophores

#### 6.1.1 D-A-D Fluorophores

Donor-acceptor-donor (D-A-D) dyes are common NIR-II fluorophores based on a symmetrical molecular arrangement of two-electron donors flanking a central electron acceptor [often benzobisthiadiazole (BBTD)] (Ding et al., 2019). This configuration lowers the energy gap—the energy separation between the highest occupied molecular orbital (HOMO) and the lowest unoccupied molecular orbital (LUMO)—which shifts the emission spectra into the NIR-II window (Qian and Wang, 2010). The emission spectra and other characteristics can be tuned by changing the donor and acceptors (Ding et al., 2019). Antaris et al. (2016) synthesized the first small D-A-D NIR-II organic fluorophore for *in vivo* imaging, CH1055-PEG (ex/em 808/1055, 8.9 kDa, quantum yield 0.3%). PEGylation was used to decrease retention in the reticuloendothelial system and increase hydrophilicity. It is water-soluble and rapidly excreted by the kidneys, giving excretion levels comparable to FDA-approved fluorophores. The authors injected it intradermally at the base of the tail in mice and showed that for FL it outperformed ICG in terms of spatial resolution and SBR despite the lower quantum yield. The quantum yield was later significantly increased to ∼5% in serum (11% if heated) by changing the functional groups from carboxylic to sulfonic acid, giving a new fully water-soluble non-PEGylated molecule termed CH-4T (Antaris et al., 2017).

#### 6.1.2 Cyanines

The polymethine cyanines (a group that includes common NIR-I dyes like ICG and IRDye800CW) have also been developed as NIR-II fluorophores, and usually show greater quantum yields than D-A-D dyes (Ding et al., 2019). Their structure (Figure 4A) can be tuned to decrease the energy gap and increase the emission wavelength (Bricks et al., 2015). These NIR-II cyanine fluorophores were developed after the promising results of imaging ICG in the NIR-II range. However, they showed significant solvatochromism-induced (rather than aggregation-induced) quenching in solution, which greatly limited their applicability. Wang et al. (2019) reduced quenching by shortening the polymethine chain length and introducing electron-donating diethylamino moieties on the terminal heterocycles, developing a series of benzothiopyrylium pentamethine cyanines (BTCs). Using BTC1070 (ex/em 1,014/1,070 nm), the authors were able to obtain very high-resolution images of draining lymphatics after intradermal injection into mice hindfeet. They were able to visualize lymphatics of 84 μm diameter with a maximum SBR of 9.42. BTC1070 outperformed ICG and the heptamethine analog IR26 imaged in the NIR-II range, in terms of brightness, resolution, and photostability (Figure 3).

#### 6.1.3 J-Aggregation

Another method of red-shifting organic fluorophores is J-aggregation. Recently, Li et al. were able to induce J-aggregation in the organic dye BODIPY by introducing a [2,2]paracyclophane (PCP) group to the *meso*-position. J-aggregation (head-to-tail packing) leads to red-shifted absorbance and emission and increased quantum yield, but H-aggregation (face-to-face packing) is usually favored in BODIPY. The J-aggregated BODIPY molecules were stabilized into nanoparticles by Pluronic F-127 and injected subcutaneously into mice foot pads. The authors visualized very bright draining lymph vessels and nodes non-invasively on an InGaAs camera with an 808 nm laser excitation source and a 1,100 nm long-pass filter.

#### 6.1.4 Aggregation-Induced Emission

As mentioned, aggregation-induced quenching is a significant problem for fluorophores, especially those with rigid structures, that emit in the NIR-II range. This can be addressed by aggregation-induced emission (AIE), where fluorophores with flexible structures, which normally have very low quantum yields, show increased fluorescence after aggregation due to intermolecular interactions causing increased rigidification (Liu et al., 2021). This strategy has been particularly useful for NIR-II fluorophores that are often disadvantaged by low quantum yield. By changing the donor groups of D-A-D dyes to give highly twisted structural backbones, Li et al. (2020b) used the AIE strategy to manufacture new organic fluorophores for lymphatic node imaging in the NIR-IIb range. One dye, in particular, HL3, which was incorporated into PEGylated nanoparticles (prepared in amphiphilic DPPE-5KPEG), showed excellent AIE features and brightness, with peak emission at 1,050 nm, which tailed to 1,600 nm [quantum yield 11.7% in NIR-II (1,000–1,700 nm) and 0.05% in NIR-IIb (>1,550 nm)]. The authors used the long emission tail to image lymphatic drainage in the NIR-IIb range to give better SBR. They injected HL3 intra-dermally into mice foot pads and imaged draining lymphatic vessels and nodes under 808 nm laser irradiation using a 1,550 nm long-pass filter. The SBR reached 5.1 for the popliteal node (compared to 2.5 using a 1,000 nm long-pass filter). Wu et al. (2019) used the AIE strategy applied to a D-A-D fluorophore by changing the heteroatom in the acceptor unit from sulfur to selenium, producing a novel NIR-II fluorophore termed BPST. To aggregate the molecules, they were encapsulated into nanoparticles (termed L897 NPs; quantum yield 5.8%) and showed an emission peak at 897 nm, with a tail extending to 1,200 nm. The authors showed clear visualization (SBR = 3.9) of popliteal and sacral LNs after intradermal injection into mice foot pads.

### 6.2 Semiconducting Single-Walled Carbon Nanotubes

Single-walled carbon nanotubes (SWCNTs) are tubular cylinders (diameter ∼1 nm) of carbon atoms with small band gaps (Table 2) (Welsher et al., 2011). They are ideal fluorescent probes due to their large Stokes shift in the NIR region and their excellent photostability (Matsuda, 2013; Zhang et al., 2021a). However, they have poor biocompatibility, which requires functionalization (Galassi et al., 2020). This is usually achieved by conjugating SWCNTs with phospholipid-polyethylene glycol (PL-PEG) (Welsher et al., 2009). Biocompatibility and quantum yield can also be improved by coating with amphiphilic poly(maleic anhydride-*alt*-1-octadecence)-methoxy polyethylene glycol (C18-PMH-mPEG) (Cai et al., 2019). Disadvantages of SWCNTs include low extinction coefficient and quantum yields (Hong et al., 2017). There is also concern regarding their possible toxicity, especially for the lung and pleura (Kobayashi et al., 2017).

Lin et al. (2019b) created PEGylated oxygen-doped SWCNTs (ex/em ∼988/1,126 nm) to obtain high contrast FL images (via a 1,150 nm long-pass filter) in mice with doses as low as 100 ng (∼4 μg/kg). Liang et al. utilized SWCNTs for a combined diagnostic and therapeutic approach in a mice model of breast cancer (Liang et al., 2014). PEGylated SWCNTs were injected into the primary tumor and the SLN was imaged at around 90 min post-injection. Photothermal ablation was made possible using the SWCNTs, and metastatic spread was substantially inhibited.

### 6.3 Rare-Earth-Doped Nanoparticles

Rare-earth-doped nanoparticles (RENPs) are core-shell structures with lanthanide ions (core) encapsulated in an inorganic crystalline host matrix (shell), which reduces quenching and enhances brightness (Table 2) (Yang et al., 2021). Doping with various other elements such as cerium (Ce^3+^) or ytterbium (Yb^3+^) can improve the quantum yield and tune the emission spectra (Yang et al., 2021). Their advantages include their large Stokes shift, narrow emission bands, and good photostability, but like other inorganic nanoparticles, their use is significantly limited by poor biodegradability and accumulation in the reticuloendothelial system (Li et al., 2019). Li et al. increased their excretion rate and decreased their toxicity by incorporating them into liposomes (Li et al., 2019). Their novel formulation (RENPs@Lips) was injected intradermally into the base of the tail in mice and the inguinal LN was imaged with a high SBR of 6.1.

The narrow emission bands of RENPs make them good candidates for multicolor imaging. Zhang et al. (2021b) developed two RENPs, NaYF4:Yb20Er2@NaYF4 and NaYF4:Nd5@NaYF4, coated with DSPE-5KPEG for simultaneous imaging in the NIR-IIb (1,530 nm, under 980 nm laser excitation) and NIR-IIa (1,060 nm, under 808 nm laser excitation) windows, respectively. They used these fluorophores to image the right and left popliteal LNs in mice after subcutaneous injection into the right and left footpad, respectively. Both PEGylated fluorophores showed good retention in the SLN.

## 7 All Wavelengths: Quantum Dots and Other Nanoparticles

Quantum dots (Qdots; diameter ∼5–10 nm) are fluorescent nanocrystals available at ∼500–1,500 nm emission (Table 2), first used for SLN detection in an animal model in 2004 (Kim et al., 2004). They are composed of a semiconductor core (most commonly Cd-Se or Cd-Te) coated with an inorganic shell (e.g., ZnS) (Kosaka et al., 2013). The shell provides a physical barrier for the optically active core, resulting in excellent bio- and photo-stability (Kosaka et al., 2013). They can be classified as type I [conduction band (where electrons are promoted to after energy input) of the shell at higher energy than the core and valence band (where electrons primarily reside) of the shell at lower energy than the core] or type II [both the valence and conduction bands in the core lower (or higher) than in the shell] (Kim et al., 2003). Today, type II QDots are generally preferred in pre-clinical research due to their NIR emission (Zhang et al., 2021a). They also display high quantum yields and a long Stokes shift, highly desirable properties for *in vivo* imaging because it allows minimal exposure times and faster frame rates (Kosaka et al., 2010). They can also be finely tuned by altering their size and structure to produce a narrow emission light across the visible and NIR spectrum (Kosaka et al., 2010). Different Qdots can be excited by the same excitation light (e.g., white light), allowing multicolor imaging (Kosaka et al., 2010). This is a particularly beneficial feature for visualizing various lymphatic drainage basins simultaneously. Kobayashi et al. (2007b) injected five different Qdots (565, blue; 605, green; 655, yellow; 705, magenta; 800, red) of similar sizes (16–19 nm) intradermally into five different sites (2x upper phalanges, 2x ears, chin). Using a spectral imaging system (Maestro *In Vivo* Imaging System, CRI Inc., Woburn, MA), they were able to simultaneously visualize the five different sets of draining LNs through the skin with a single excitation source, even though a double dose of Qdot 565 was required due to its weak emission within a high autofluorescence background. In a separate study, they performed real-time *in vivo* visible multicolor fluorescence imaging with a modified real-time fluorescence imaging system (FluorVivo, INDEC BioSystems, Santa Clara, CA), using an excitation band-pass filter of 450–490 nm and an emission long-pass filter of 520 nm and obtained comparable results (Kosaka et al., 2009).

A disadvantage of currently-used visible wavelength Qdots is that the excitation light commonly induces autofluorescence, reducing the SBR. Spectral unmixing, using the Maestro *In Vivo* Imaging System mentioned earlier, is a technique to remove presumed autofluorescence, but it requires long acquisition times and manual postprocessing, making it difficult to obtain real-time images (Kosaka et al., 2010). This issue can be minimized by using a longer excitation wavelength, which reduces autofluorescence while obtaining a shorter emission wavelength. At the level of the imaging device, this has been achieved using MPLSM mentioned earlier. It can also be done with upconverting nanoparticles (UCNPs), which are RENPs that can convert the energy of two serially absorbed photons to emit light at shorter wavelengths than the excitation light (Chatterjee et al., 2008). Kobayashi et al. (2009b) demonstrated the utility of visible UCNPs for SLN imaging by injecting NaYF4:+Yb and Er (UC-Green) [with two narrow emission peaks at 550 and 670 nm (green)] intradermally into the necks of mice. Real-time fluorescent imaging was performed with 980 nm laser excitation, which they showed produced no autofluorescence. The draining LN was clearly visible with a high SBR in real-time, but only with the skin removed as emission was in the visible range. Autofluorescence can also be eliminated by using bioluminescence resonance energy transfer Qdots (BRET-QDs). BRET-QDs do not require excitation light as they self-illuminate when the co-administered substrate coelenterazine reacts with luciferase, which is conjugated on the BRET-QD (Kosaka et al., 2010). This technique has also been used to visualize draining LNs at a wavelength of 655 nm (Kosaka et al., 2010). However, both UCNPs and BRET-QDs show weaker emission light intensity than Qdots, and thereby require slower frame rates (Kosaka et al., 2010).

The inherent brightness of visible wavelength Qdots allows LNs to be visualized through the skin but because of their superior tissue penetration, NIR Qdots are most often used. Kim et al. non-invasively visualized SLNs after intradermal injection in mice and pigs in the NIR (emission 840–860 nm) using type II QDots that had a polydentate phosphine coating to increase solubility and stability in serum (Kim et al., 2004). The QDots showed no photobleaching and actually displayed some photo brightening. They had diameters of 15–20 nm and a protein-equivalent size of 440 kDa, which was adequate for lymphatic uptake and retention in the node. NIR-II Qdots are also now in use for lymphatic imaging. Tian et al. (2020) developed a method of dual NIR-II/SWIR imaging using two different probes that targeted both the draining SLN and metastatic cancer cells simultaneously. The NIR-II/SWIR ranges are ideal for multicolor imaging as the broad range allows the use of fluorophores that do not overlap in emission spectra. The SLN was imaged in mice at >1,500 nm after intratumoral injection or intradermal injection into footpads of ultrabright PbS/CdS (core/shell) Qdots with a dense polymer coating (diameter < 20 nm) (Figure 3). Furthermore, the Qdots were conjugated with the T-cell-specific antibody anti-CD3 to enhance SLN uptake and retention and alleviate safety concerns (see the following). The SBR of the Qdots was 1.26 at 9 mm depth, compared to 1.07 for ICG at 5 mm depth. They also showed excellent photostability and quantum yield (6% in water). Metastatic tumor cells were targeted by intravenous administration of a PEGylated tumor-seeking D-A-D dye, IR-FD, and imaged at 1,100–1,300 nm.

Unfortunately, both Qdots and UCNPs have potential toxicity to humans (Kosaka et al., 2010) and so are unsuitable for clinical use. Retention of QDs within the reticuloendothelial system for up to 2 years has been documented in mice (Kim et al., 2004). This retention not only limits clinical translation but also limits longitudinal studies in animals (Proulx et al., 2013). However, because of their superior SBR, it is proposed that UCNPs can be used at lower doses, thereby somewhat alleviating toxicity concerns (Kobayashi et al., 2009b).

## 8 Future Directions

Despite the widespread successful use of ICG for fluorescent lymphatic imaging, there is still scope for further development of fluorescent dyes for lymphatic imaging and there are some exciting prospects in this regard. Future research directions fall under four main categories: 1) fluorophores that emit at longer wavelengths, 2) directed targeting to lymphatic vessels and nodes, 3) improving non-invasive functional imaging, and, most importantly, 4) addressing barriers to clinical application. As mentioned, NIR-II dyes and UCNPs represent opportunities for fluorophore development. Rare earth UCNPs were previously too large for optimal lymphatic distribution (>100 nm) (Kobayashi et al., 2009b), but newer developments have now brought their size down to as low as 10 nm (Zhang and Xu, 2019). Because of their excellent penetration and SBR, these hold significant potential for clinical applications. Molecules that fluoresce in the SWIR region also hold these potential advantages and are under development (Schnermann, 2017), although heat damage is a possibility at these wavelengths. In addition, specific targeting of antigens on lymphatic vessels or nodes at longer wavelengths, or lymphatic-draining immune or tumor cells, represent areas of future promise. Similarly, fluorophores that fluoresce only in response to activation by a lymphatic vessel or node, or under certain conditions such as inflammation (Li et al., 2020c), hold great promise for increasing the specificity and applicability of lymphatic-targeted fluorophores. With the recent development of a wide range of NIR-II fluorophores, future study can be directed toward non-invasive functional lymphatic imaging, especially lymphatic contractile function, in an effort to understand the additional information that can be gained from imaging in the NIR-II range compared to NIR-I and optoacoustic imaging. Last, future research should be directed toward molecules with decreased toxicity and increased solubility to improve clinical applicability. It is unfortunate that toxicity concerns have particularly limited the applicability of Qdots, which otherwise hold promise (Hama et al., 2007). High-quality fluorescent imaging is also crucially dependent on the imaging device, and the development of smaller and cheaper imaging devices would be highly beneficial for more widespread clinical use. Despite the many fluorophores under development, approval for human use and clinical translation still present significant barriers that require ongoing research.

## 9 Conclusion

FL holds many advantages over other imaging modalities, including price, access to equipment, lack of radiation exposure, spatial resolution, and ease of use. Because of these, FL is in widespread use in both pre-clinical and clinical settings. The most appropriate fluorophore depends on many factors, such as the indication (clinical vs. pre-clinical and SLN detection vs. functional imaging), tissue and depth of imaging, safety profile, equipment availability, etc. Non-invasive imaging of dermal lymphatics and superficial LNs has rapidly improved over recent decades. The most common route of administration is an injection into the interstitium (i.e., intradermal, subcutaneous, submucosal, intratumoral, intraparenchymal), where targeted lymphatic uptake occurs based on interstitial and tracer properties, especially size and charge of the fluorophore and/or conjugate. The lymphatic system can also be accessed *via* enteral delivery to mesenteric lymphatics, intraperitoneal delivery, or *via* direct injection into a LN or lymphatic vessel, however, these latter methods are likely to require surgical exposure to visualize the vessels or are invasive and damaging to perform in the case of the latter. Visible wavelength fluorophores have been used for surgically exposed lymphatic vessels and nodes and for FML, where the most superficial dermal lymphatics are visualized with a microscope. However, because of their superior depth penetration, NIR fluorophores have been more widely used for non-invasive imaging. The most commonly used NIR fluorophore is ICG, which is FDA-approved and has been in clinical use for decades. However, it has certain limitations (low quantum yield, instability in solution, uncertain SLN retention, lack of a functional group, and limited high-resolution imaging at depths) that have motivated researchers to search for new ICG formulations or new lymphatic-targeted fluorophores for clinical translation. There are many new NIR-I and NIR-II fluorophores under development to address these issues. Most rely on the addition of molecular constructs to aid lymphatic targeting, such as PEG or lymphatic-, immune cell-, or tumor-targeted antibodies (the latter has allowed the use of intravenous administration, where the fluorophore accumulates in metastatic LNs). Fluorophores that emit at longer wavelengths are of particular interest for non-invasive lymphangiography due to even greater depth penetration. The progression of these newer fluorophores to clinical use is set to greatly improve the imaging capability of FL.
